# Urea-Based Eutectic Mixtures as Green Plasticizers for Starch, Other Polysaccharides and Proteins

**DOI:** 10.3390/molecules31183244

**Published:** 2026-09-14

**Authors:** Magdalena Zdanowicz

**Affiliations:** Center of Bioimmobilisation and Innovative Packaging Materials, Faculty of Food Sciences and Fisheries, West Pomeranian University of Technology Szczecin, Janickiego 35, 71-270 Szczecin, Poland; mzdanowicz@zut.edu.pl

**Keywords:** biopolymers, deep eutectic solvents, molten mixtures, plasticizers, starch

## Abstract

Urea (U), known as carbamide, is a cheap, ubiquitously available diamide widely used in the chemical industry. In the last two decades, this polar compound has become a component of novel green media: deep eutectic solvents (DESs), used, for example, as catalysts, solvents, modifiers, and plasticizers. Due to their physicochemical properties, they can replace molecular ionic liquids as a cheaper, more environmentally friendly alternative. Urea can be both a donor and an acceptor of hydrogen bonds, thus giving a wide range of possibilities for novel media design. Formation of H-bonding in eutectic mixtures has a lot of advantages compared to pure urea or conventional media, especially as a modifier for biopolymers. U-based DESs can be used as functional plasticizers for many abundant biopolymers like polysaccharides (especially starch, but also cellulose derivatives, agar, agarose, alginate, and chitosan) and proteins (e.g., gelatin), leading to the formation of their thermoplastic derivatives that could substitute some oil-based plastics in the future on an industrial scale.

## 1. Introduction

### 1.1. Introduction to Urea

Urea, known also as carbamide (the simplest amide of carbamic acid), in everyday life is mostly recognized as a fertilizer. More than 90% of its production is aimed for agriculture, and the global production in 2024 exceeded 200 million metric tons [1,2,3], with China as a leader responsible for ca. 30% of the world supply. Urea is rich in nitrogen (46% of molecular mass), and the main reason for its fertilizing activity is the release of plant-available ammonium during enzymatic (urease-driven) decomposition in the soil [4]. Other applications of urea are urea–formaldehyde resin production, moisturizing additives for skin care products, and barbituric acid manufacturing [1].

Urea was first isolated from urine in 1773 in a very impure state. However, its presence was recognized much earlier by Van Helmont, who assumed that the “urine salt” originated from the digestion of substances (1577–1644). Almost 200 years later, Hilaire Martin Rouelle proved that this compound is rich in nitrogen. The first isolation of pure urea is dated 1814 [5]. The synthesis of urea turned out to be a pivotal event, contributing to the development of organic chemistry as a discipline and the abandonment of the vitalism path [5,6]. Nevertheless, urea formation was non-intentional and occurred during Wöhler’s attempt to produce ammonium cyanate [6]. Opposite to the chemist’s predictions, unexpected “vegetable salt base” appeared according to the following reaction [7]:

AgNCO + NH_4_Cl → (NH_2_)_2_CO + AgCl (1)

Then, 40 years later, A. Basarov obtained this simple carbamide by heating ammonium carbamate under pressure:

2 NH_3_ + CO_2_ → NH_2_COONH_4_ (125–250 bar)  → NH_2_COONH_4_ → (NH_2_)CO + H_2_O (170–220 °C) (2)

Reaction (2) is the basis for large-scale production of urea. Jozef Meessen, in [7], showed and explained the production processes.

### 1.2. Brief History of Urea and Its Deep Eutectic Solvents

Urea is a colorless, odorless, and highly soluble in water solid, which melts at 133–135 °C. Chemically, urea is a very weak base, but due to the lack of ubiquity of amides and reactivity of esters, it is considered barely reactive [8], which, as we will see further, can be an advantage. As a hydrogen-bond donor (HBD), urea and its derivatives have found application as organocatalysts and anion transporters [8]. Urea (and its “cousins,” including thiourea and dimethlurea) as (not only) HBD can form physical mixtures, such as molten mixtures or molten salts [9,10], and deep eutectic solvents [11]; substituted ureas have found utilization as, for example, scaffolds in supramolecular chemistry [8]. Molten urea can also be used as a reaction medium, for example, in cellulose phosphorylation [12,13], and Sha et al. [14] used DES as a reactive medium for starch to obtain its cationic derivative. Imperato et al. [9] prepared highly polar molten systems based on mono- or disaccharides, U, or DMU and chloride salts with melting temperatures in a range of 65–89 °C and applied them as reaction media for hydrogenation reactions.

The urea played a key role in the appearance of the term “deep eutectic solvents,” which was introduced for the first time by A. P. Abbott and coworkers in 2003 [11]. The authors, relying on certain previous works from three decades, had found that urea can form eutectic mixtures with alkali metal halides, and their work presented new systems based on choline chloride (CC) and other quaternary ammonium salts (Table 1).

Thus, their work was trailblazing for DES, but in fact, urea-based eutectic systems were already known half a century ago. In 1972, Tomas and Belyanin [15] presented a study on the utilization of a eutectic consisting of 55% urea and 45% soda for the pretreatment of gray cast to improve wear resistance. Nowadays, some mixtures are called “DES,” but they already existed before 2003; for example, U:sodium acetate trihydrate as DES was used as reaction media [16], whereas Wada, Kimura, and Yamamoto presented this eutectic mixture for latent heat storage in 1988 [17]. Going back to Abbott’s work [11], at first, they called their systems eutectic mixtures/liquids and classified them as ionic liquids. Breaking down the term into individual words: “deep” came from great drop of freezing point for the eutectic in comparison with the individual components; “eutectic” is a root indicating the physical mechanism, and “solvent” is due to these systems exhibiting dissolving activity influenced by H-bonding toward, for example, salts, metal oxides, aromatic acids, and amino acids, similarly to most of the molecular solvents and ionic liquids.

The main and most popular definition of DES is based on the difference in melting temperature, but Abbott and coworkers determined the freezing point. Some natural DESs (NADESs) do not exhibit T_m_, only glass transition; thus, generally, DES can be defined as a system where the phase transition temperature (T_p_) of the mixture is much lower than T_p_ of its individual components. The mechanism is based on hydrogen bond donor (HBD) and hydrogen bond acceptor (HBA) interactions [11,18], so systems composed, for example, only of HBD, such as mixtures of polyols or sugars, should be classified as low-transition-temperature mixtures—LTTM [19,20]. The term “DES-like” was proposed for systems that share some physicochemical properties with mixtures that do not form typical eutectics, for example, mixtures where one of the components is already liquid [21]. Another issue is that DES sometimes are labeled as ILs; however, considering mainly the preparation methods, they should be allocated in a separate category, such as a subgroup of LTTMs [22]. Moreover, due to the simple method of DES formation and the mechanism responsible for the drop in transition temperature (which is presented in Section 1.3), DES preparation should not be called “synthesis” (which has surprisingly been used quite often) due to the lack of chemical reaction [22]. DESs are greener, cheaper, and easier to prepare than ILs. Preparation of U-based DESs mainly involves simply mixing components in a certain ratio at elevated temperature; thus, “synthesis of DESs” is actually not a proper term for DES preparation [22].

Taking into account that urea and its DESs, such as U:CC, form H-bonds but are barely reactive, they are good solvents or reaction media both for small molecules as well as for macromolecules [23,24], replacing less green ILs. The yield of O-acetylation reaction of monosaccharide conducted in U:CC 2:1 was less than 5%, whereas in CC:ZnCl_2_ 1:2, it reached 96% [25]. Notwithstanding, urea as HBD can form bonds with solvents or reaction media, increasing the catalytic activity of the system, for example, in polyester glycolysis [22,26,27]. U-DES as a polar system can dissolve many compounds with similar character, even some biomacromolecules [23,24]: polysaccharides, such as starch [28,29], guar gum [30], agar [31], or proteins [32,33]. However, not all biopolymers are susceptible to dissolution by DESs. U-DESs dissolving activity can be tailored by, for example, the type of components or water addition, which can facilitate or inhibit dissolution. Water addition to DES facilitated gelatin dissolution [31] and pectin extraction [34], whereas for starch, it shifted its dissolution toward gelatinization [35].

### 1.3. Why Do Urea Forms Eutectics with Certain Compounds?

**Figure 1:**
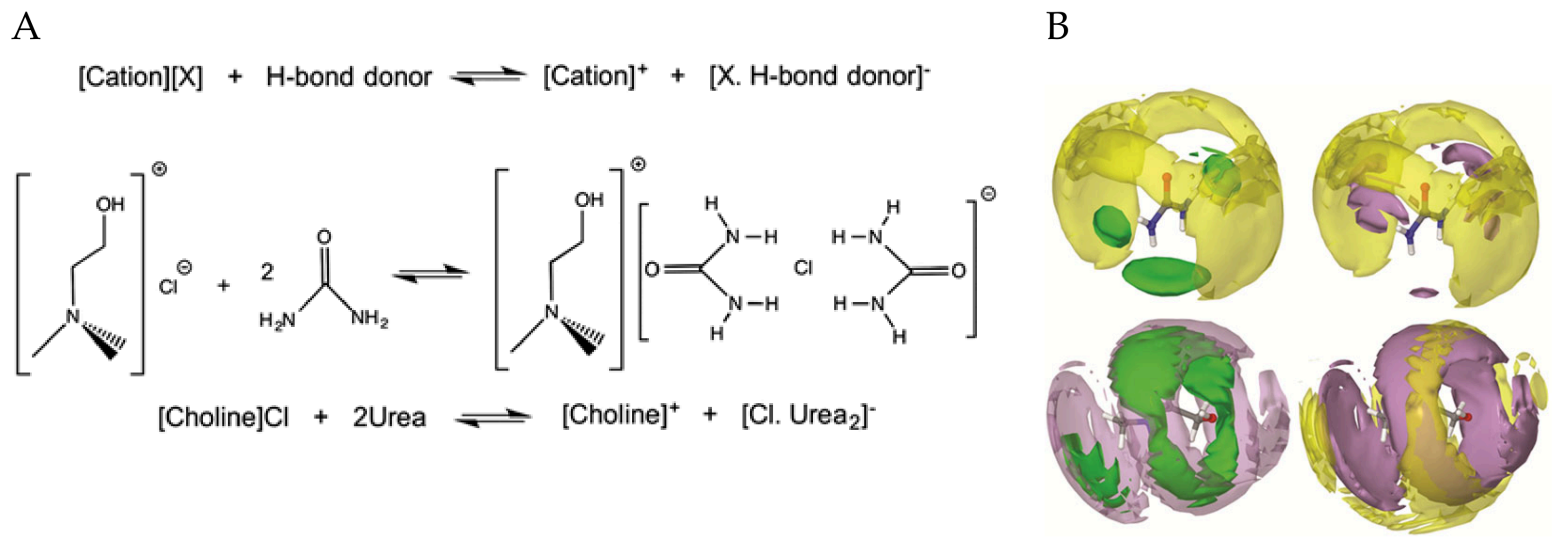
Schemes of structures in choline chloride:urea DES: (**A**) Possible complex formation. The figure is reprinted (adapted) from Ref. [39]. Copyright, 2016, Royal Society of Chemistry; (**B**) Probabilistic 3D structures. Yellow surfaces depict choline^+^, purple is for U, and green is for Cl^−^. Reprinted (adapted) from Ref. [41]. Copyright, 2016, Royal Society of Chemistry.

The most well-known DES is Abbott’s team’s mixture of choline chloride and urea, which, in a molar ratio of 1:2, is liquid at ambient temperature. This mixture is called “reline.” In their communication article, which has accrued almost 6000 citations, hydrogen bonding engaging ions from HBA and NH_2_ protons was indicated as the reason for freezing depletion [11]. The role of H-bonding formation between quaternary compounds, such as CC or betaine, was confirmed by NMR analysis [11,36,37]. Among HBA ions, anions play the main role in complexation between the components [38,39,40]. Sun et al. [38] presented the role of U in mixtures with CC. Based on molecular dynamics simulation, the study showed that the presence of U significantly changed the density distribution of the ions, disrupting long-range ordered structure and cation–anion interactions in the quaternary ammonium salt. The study found that in Cl^−^ of HBA preferred to be positioned with hydrogen atoms associated with largely electronegative OY (Scheme 1 in [35]) and that AN^+^ led to more positive H atoms associated with OY and CA atoms. An increase of U content in the mixture disturbed anion density distribution near hydrogens from carbon associated with AN^+^. The enhanced interactions between U and ions led to a decrease in melting point. The continued investigation of U:CC mixture interactions by Ashworth et al. [39] confirmed that U can act as a spacer, separating ions in the eutectic systems. According to the authors, U:CC can be considered as the “alphabet soup” of many different types of hydrogen bonding. Even though, considering the chemical structure, the formation of symmetrical [Cl(U)_2_]^−^ complexes has been intuitively suggested (Figure 1A), their anionic charge is delocalized, and other forms of competitive complexes’ presence, that is, U[choline]^+^ with a strong H-bond in OH∙∙∙O=CH. Thus, they proposed U[choline]^+^ ∙[Cl(U)_2_]^−^ as the favored structure. They remarked that there are a variety of interactions where urea can act both as HBA and as HBD. This can be a reason why U can form LTTMs/DESs with sugars or polyols [29].

Hammond et al. [41], relying on spatial density functions modeling, proposed probabilistic 3D structures of reline components (Figure 1B). Each chloride anion is solvated by an average of two U molecules with H-bonding of 2.2 Å, which corresponds to the results obtained in another work [39], and U is ordered around OH groups of choline with many possible conformations forming a radially layered structure.

**Figure 2:**
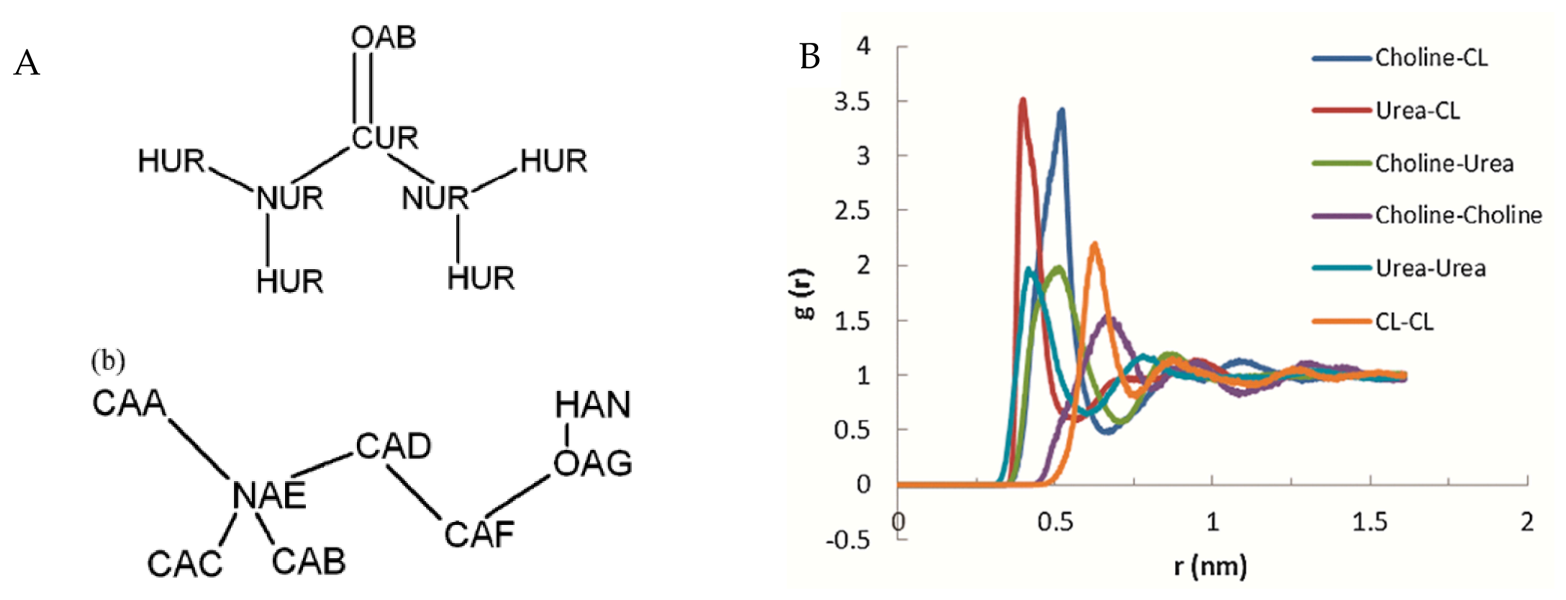

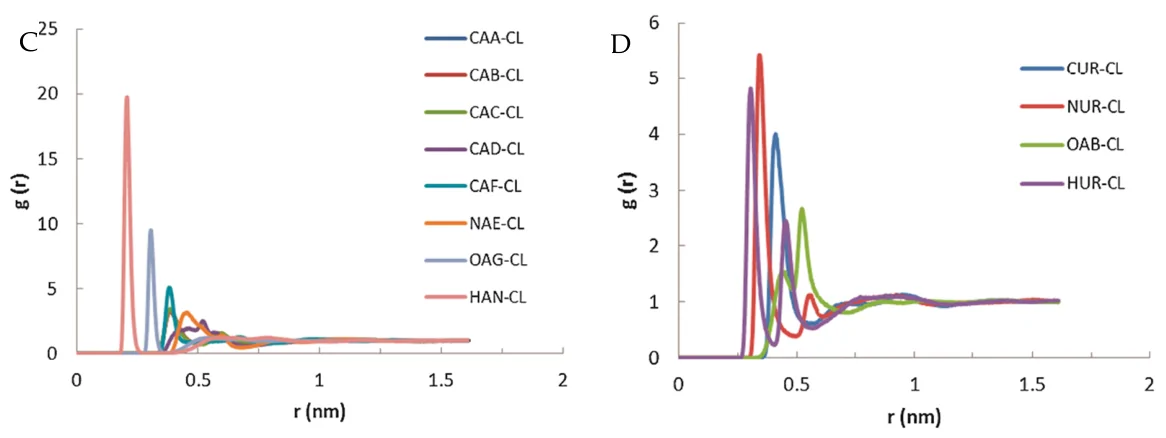
(**A**) Chemical structures of urea and choline molecules; center-of-mass radial distribution functions (RDF) between (**B**) U and CC molecules; (**C**) atom–atom RDF between choline and chloride (CL); (**D**) atom–atom RDF between urea and chloride. The figure is reprinted (adapted) from Ref. [42]. Copyright, 2016, Royal Society of Chemistry.

The microstructure of reline and atom–atom interactions was calculated in works [41,42]. Figure 2 shows the center of mass between CC and U molecules in their DES, as well as atom–atom interactions determined based on the radial distribution function [42].

The sharp, strongest peaks in the graphs represent the interaction of Cl-U (Figure 2B) and Cl-choline (Figure 2B). The broader and weaker peaks of U-U reflect that after mixing of the composition, the interactions between anion and U can provide enough energy to overcome the lattice energy of the solid particular components, counterbalancing interactions. A weak peak for U-choline shows that U molecules interact preferentially with Cl rather than with choline. Sharp peaks for Cl—with choline (Figure 2C)—and U (Figure 2D) show that chloride mainly interacts with U through a hydrogen atom and with choline through the hydrogen atom belonging to its hydroxyl group.

The explanation for the role of water in the molecular structure of DES can be found in many works [43,44,45,46,47,48]. Dai et al. [49] indicated that natural DESs (composed of metabolites like sugars, carboxylic acids, or amino acids) form supramolecular structure with less than 50 *v*/*v*% water (higher water content led to the disappearance of the interplayed interactions). Similar behavior was also observed for U-DES. Zhekenov et al. [44] performed molecular dynamics simulations of CC-DES with different HBDs, including water presence. H-bonds between HBD, choline^+^, Cl^−^, and water decreased with increasing water content. In reline, H-bonding between U and water molecules increased more noticeably than for eutectics with polyols. Less than 30% of the water molar fraction, which is 5% by weight, can be absorbed into the molecular network without impact on ion diffusion. Di Pietro et al. [47] evaluated the influence of water on U mixtures with two choline salts: chloride and acetate. The study found that the type of HBA anion significantly affects the molecular structure of DES and DES/water system. Acetate anions can make U more prone to interact with water compared with chloride anions, whereas CC in DES can retain structure, and the mutual interactions change more gradually. The competitive interaction of DES components with water can not only affect DES physicochemical properties (see Section 1.4), but also the processes on macromolecules, especially their solubility, stability, and, in the case of proteins, denaturation.

### 1.4. Physicochemical Properties of U-DES and Their Role as Plasticizers

The physicochemical properties of U-DESs, such as phase transition temperature, viscosity, density, ionic conductivity, or surface tension, depend on the molar ratio of the components, their chemical structure (e.g., anion type in quaternary salt, length of alkyl chains, and type of substituted groups), and water content [50]. Due to this review focusing more on U-DES application for biopolymers, detailed characterization of DESs is omitted except for some features important for the processing and further application, such as polarity, thermal stability, viscosity, and toxicity.

Polarity, the driving force of solubility of DESs, was determined using solvatochromic optical responses. The studies indicated that the results were influenced by electronic absorbance probes and used for the tests [51,52]. Reline was slightly more dipolar than the CC mixture with glycerol, ethylene glycol, and malic acid (based on the pyrene polarity scale) [51]. Water can easily penetrate DES network solubilizing U molecules, which was confirmed by studies employing molecular modeling. Ions and U are strongly bonded to each other but become weaker and diffuse more freely with water addition, which can be reflected in ion conductivity and viscosity. The addition of 25% of water led to separation of the components. Patra et al. [53] studied the influence of U content in DES with hydrated salts on their polarity. Dipolarity (π) decreased, whereas HBA basicity increased. Generally, the high polarity of U-DES makes them compatible with biomacromolecules, facilitating their processing.

The toxicity of DES, again, depends on the type and ratio of DES components, the method of dosing, and the studied model [21,54,55,56,57,58,59,60]. In the literature, one of the phenomena related to the issue is higher cytotoxicity of DES than their individual components [57,60]; however, the results lacked a control group to exclude other possibilities of a toxic effect [21]. Moreover, determining the MIC—minimum inhibitory concentration—requires a dilution of the tested substances, so DES structure can be disrupted, affecting the final results. Juneidi et al. [58] performed toxicological and biodegradation tests comparing CC-DESs with different HBD and their individual components. MIC tests were carried out using *Aspergillus niger* strains and the lethal concentration—(LC_50_)—was evaluated on *Carpinus carpio* fish. The results for CC:U with comparison to other, most common DESs and individual components of reline are listed in Table 2. The data revealed that DESs, according to the acute toxicity rating scale by the Fish and Wildlife Service (FSW), are relatively harmless. Urea presence led to a decrease in MIC values, much lower than CC-DESs with alcohols. It can be seen that urea plays a key role as a partly inhibiting agent because the MIC for reline (138 mg/mL) is only slightly higher than for pure U, despite the dilution with CC. Results indicated that these most common DESs do not exhibit antibacterial properties, so they do not bring extra functionality as additives. To increase antimicrobial properties, DESs made with zinc salts [56,58] or nanoparticles, for example, of silver, can be introduced [61]. However, using such DESs as additives in polymer materials would require deep investigation of migration and cytotoxicity, which has not been done so far in an extensive manner. DESs tested on mice (dose 1000 mg/kg) did not damage DNA but exhibited some metabolic effects [59].

Biodegradability tests conducted in closed bottles showed that U:CC exhibited a bit lower biodegradability than G:CC, reaching 85% of degradation degree [58].

Other properties, such as viscosity, thermal stability, and water contact angle of U-DES, can be found in numerous works [28,36,50,62,63,64]. The viscosity of DES is a key factor for macromolecule processing methods, such as extraction and dissolution, but it is discussed for plasticization. Low viscosity (reline exhibits very low viscosity, lower than CC:polyalcohols) can facilitate mixing and DES diffusion, but this can be important for thermocompressed systems.

It is also worth mentioning sources for DESs preparation. Very often, these systems are presented as “natural origin.” However, reline—the most popular one—is composed of the quaternary ammonium salt, which is synthesized industrially. Isolation of choline from natural sources might introduce issues with efficiency and cost [21]. Observing the fertilizer market, it can be found that U production depends on fossil fuels. Life cycle assessment (LCA) for different DESs and common organic solvents as reaction media was presented in [65]. The authors showed that the environmental profile of reline was compared with that of common organic solvents like methanol, ethanol, dichloromethane, and ethyl acetate based on their utilization as a solvent for alcohol oxidation. On the other hand, the LCA of reline as a CO_2_ capture system showed an environmental impact reduction of approx. 35% compared with the Selexol-based process. In comparison with choline CC:monoethanolamine, reline requires less energy and results in lower adverse environmental impacts [66]. This shows that DESs require further, deeper LCA studies in the context of their applications.

Due to the wide range of composition options of DES formation, they can be customized for certain applications or with different properties, for example, viscosity, conductivity, acidic, or basic character. We can compose therapeutic, supramolecular, or hydrophobic DESs. Urea-based eutectic systems are polar and hydrophilic, and due to their low or lack of reactivity, H-bonding formation are suitable for the treatment of molecules and macromolecules with more polar character. U-DESs have been utilized not only as solvents for gels or eutectogels forming [30,67] or as reaction/extraction media [14,33,68], but also as polymer modifiers, for example, as plasticizers or agents, which alter surfaces or bulk of the materials, leading to, for example, changes in surface tension or crystallization degree [22]. Plasticizers are very important additives in polymer technology, affecting processing and practical properties of the products, such as increasing flexibility and workability and reducing manufacturing friction. H-bonding formation with OH or NH groups of plasticizers with biomacromolecules and its interposition between polymer chains is a principle of plasticization. Plasticizers are divided into external plasticizers that are compounds of low vapor pressure, which interact with the polymer without a chemical reaction, and internal plasticizers, which are a part of the polymer molecule. Examples of these groups include a second monomer in a copolymer, for example, with a longer alkyl group, a substituted group on the main chain, or a grafting oligomer/polymer, making the structure less ordered. Another classification is based on solvent power: primary—“true plasticizers”—which are solvents for both the amorphous and the crystalline fraction of polymer, and secondary plasticizers, which do not dissolve the polymer (e.g., softeners) [69].

They can be crucial plastic components but can also be controversial due to their toxicity and migration from the matrix (e.g., phthalates). Therefore, nontoxic green plasticizers have been developed, for example, bio-based esters, plant-origin polyphenolic systems or hydrophobic DESs for polylactide, and other polyesters, such as poly(vinyl chloride) [70,71,72]. The prediction for the global plasticizer market in 2027 is an increase to 22.5 billion USD [73]. Taking into account the green character of most U-DESs and considering the trend of developing more environmentally friendly non-oil-based materials, this work focuses on natural-origin, abundant sources: polysaccharides and proteins as biodegradable, nontoxic polymers that can be applied in many areas of industry accustomed to daily life: food, pharmaceuticals, or cosmetics.

## 2. U-DES as Plasticizers for Starch and Their Composites

Starch is one of the most abundant polysaccharides, biosynthesized by plants as a storage material. This polymer with anhydroglucose units consists of two fractions: helical amylose and branched amylopectin. Their ratio depends on botanical origin and affects physicochemical properties, such as gelatinization temperature or type of crystallization. Quite strong H-bonding between hydroxyl groups gives water-insoluble character to the polymer but gelatinizes in aqueous dispersion during heating. This system, after water evaporation, forms brittle materials. Pure starch as a base for thermoplastic bioplastics processed by more industrial methods, that is, thermoformation by, for example, extrusion, is insufficient due to lack of tendency to form a melt under processing conditions, which is a result of H-bonding and T_g_ near to degradation temperature. Starch plasticizers are polar compounds that can disrupt hydrogen bonds in starch and form new ones between their molecules and the polysaccharide. The most popular plasticizers are glycerol and other polyols like sorbitol, amines, and amides; among them, U is the most common. Although they are cheap and widely available, they exhibit some drawbacks: glycerol migrates in the matrix [74,75], U can recrystallize [76,77], and amides can be toxic. During the IL boom, they were widely investigated as starch plasticizers, followed by successful results, but their green character is disputable. Moreover, ILs for mass production scale are too expensive. Due to DES exhibiting similar properties to ILs, they can also be examined as media for starch treatment, firstly as solvents, then as plasticizers [23,78,79]. Urea-based DES used for starch plasticization is listed in Table 3.

In the literature, the first report on starch treatment with DES (U:CC and U:CaCl_2_) was presented by Biswas et al. (2006) [80]. Then, in 2010, Shamsuri and Daik [81] used protic U:IL; however, in both works, DESs were presented as solvents.

**Figure 3:**
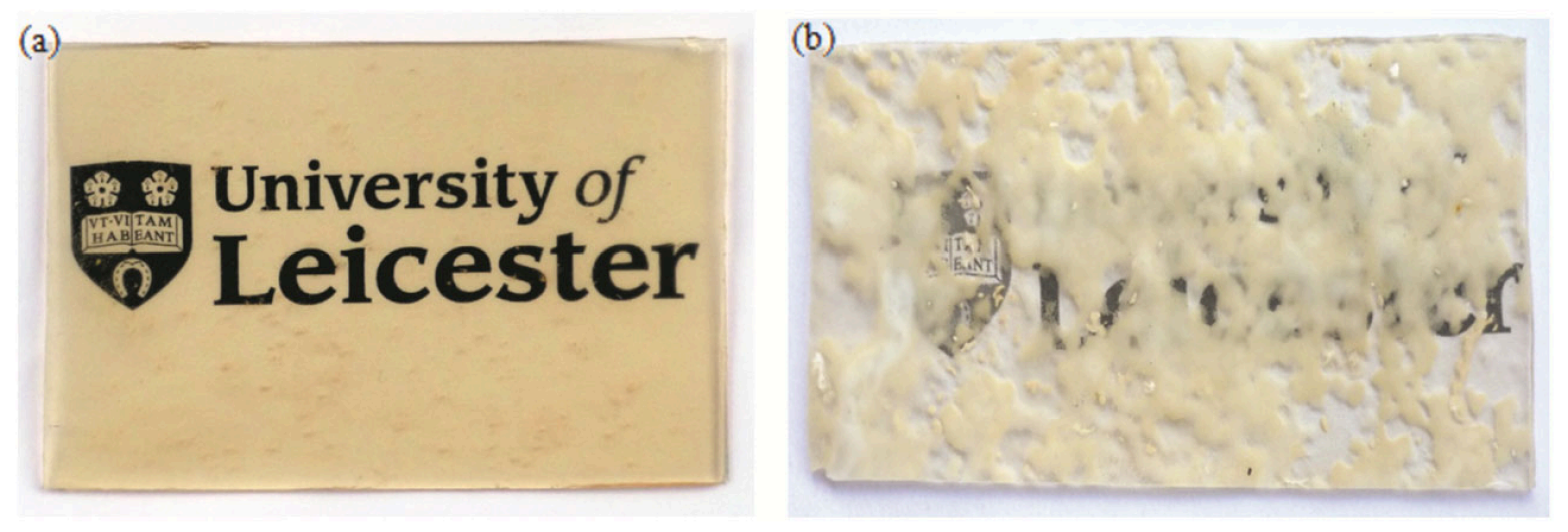
Transparent sample prepared from compression-molding of a 5.05:1:1.16 mixture of starch, urea, and CC (**a**); the sample prepared with corn starch: urea 7:3 wt ratio (**b**). The figure is reproduced from Ref. [83]. Copyright 2012, Royal Society of Chemistry.

Zdanowicz and Spychaj [82], for the first time, attempted starch plasticization using CC:U and compared the effects with [AMIM]Cl and CC:carboxylic acids. Starch/DES premixtures were thermocompressed in a laboratory hand press with a pressure of 20 MPa at 140 °C for 10 min. For the tests, two types of potato starch were used: dried and moist (14% of moisture), indicating that the presence acted as co-plasticizer facilitating starch transition. Abbott, the father of CC:U DES, was also the first to use choline chloride and U for corn starch (dried) plasticization via extrusion (120 °C, 120 rpm). The results were compared for only thermocompressed systems (140 °C, 10 min). For the thermocompression, TPS was also processed with pure urea. Here, I need to highlight that CC and U were added separately (not as liquid), but the premixture had been conditioned at 70 °C for 1 h before molding, leading to the creation of a transparent TPS film. However, TPS processed exclusively with U, despite being even more elastic, exhibited U recrystallization (Figure 3). In the case of DES, H-bonding formation between CC and U occurred, which inhibited U recrystallization. Better mechanical properties were achieved for extruded materials. Additionally, the authors indicated that TPS/CC/U can be remoldable. To prevent moisture absorption by highly hydrophilic TPS (CC is very hygroscopic), the films were coated with PLA [83]. Then, Leroy et al. [84] extruded corn starch with 20% of different plasticizers with microcompounder, examining parameters during processing. The differences between values of specific mechanical energy (SME) and viscosity were as follows: [BMIM]Cl < CC:U 1:2 < CC:G 1:2 < G. A similar trend was observed for the tendency to undergo retrogradation. Additionally, the authors compared the mechanical properties of TPS and their blends with zein (90:10). The trend of elongation at break (EB) changes was the same as above, and tensile strength was opposite. Moreover, in the case of IL and DESs, zein presence did not negatively affect the mechanical properties, whereas for G, they deteriorated after protein addition.

**Table 3:** U-DESs as plasticizers for starch and their composite. Chronologically listed works.

U-DES	Type of Starch	Method of Preparation	Characterization	Remarks	Ref
U:CC 2:1	potato	thermo- compression	Dissolution (microscopy), rheometry of premixtures followed by XRD	Comparison with IL and DES CC: carboxylic acid	[82]
U:CC 2:1	corn	heating, thermocompression, extrusion	SEM, DSC analysis for premixture and extrudate, quartz crystal microbalance experiments, conductivity	CC and U added separately to starch. Comparison of TPS with pure urea. Conducting materials	[83]
U:CC 2:1	corn corn + zein	extrusion in microcompounder and thermocompression	SME and viscosity during thermoplasticization, DMTA, XRD, LSM, mechanical tests	Comparison with ILs and other DESs and blends with zein (10 wt%). Investigation of plasticizer type on processability	[84]
U:CC 2:1	corn	extrusion	Mechanical tests (thermal characterization for G:CC)	Remoldable material, comparison with other HBD: G and EG in DES. Processing conditions studied only for G:CC	[85]
U:CC 2:1	corn + electrolite	casting	Impedance spectroscopy, ionic conductivity, SEM, FTIR	Different DES content, gel formation for more than 40% of DES	[86,87]
U:CC 2:1 U:CC:G 2:1:2 U:CCit:G 2:1:2 U:CCit 2:1 U:CC:S 1:1:1	potato and hydroxypropyl oxidized	casting	Mechanical tests, WVTR, WCA, moisture content, solubility, FTIR, OTR for selected samples	Different DES content and left at RT to dry (few days). Comparison of introducing DES and their component separately. CCit as crosslinking component	[88]
U:CCit:G 2:1:2	potato hydroxypropyl oxidized + MMT/MCC/tannin	casting	Mechanical tests, WVTR, WCA, moisture content, solubility, FTIR, OTR for selected samples, polarizable microscopy	Filler type and content highly affected mechanical and barrier properties	[89]
U:CC 2:1	corn + zein	extrusion	Confocal microscopy, XRD, mechanical tests including finite element modelling, representative elementary size	Study on influence of different plasticizers (ILs, G, and DESs) on mechanical properties and microstructure of the blend	[90]
U:CC 2:1	potato + U-MMT	extrusion	Mechanical tests, DMTA, ARES rheometry, XRD, WCA, thermal conductivity	Comparison with TPS/IM:CC. Thermal conductivity for TPS/U:CC 0.235 W/mK, decreased with U-MMT addition. High EB value (245%)	[91]
U:CC 2:1	potato + wood fiber	thermo- compression	Mechanical tests, DMTA, LSM, WCA, FTIR	Study of pretreatment of filler with DES. High content of the filler in TPS. Partial “leaking” of lignin into TPS with DES	[92]
U:CC 2:1 U:CAc 2:1 U:CLa 2:1	potato	thermo- compression	Dissolution tests (polarizable microscopy, DSC), ARES rheometry of premixtures, mechanical tests, DMTA, FTIR, TGA, solubility, swelling degrees	Comparison with G and ILs. Study of influence of different anions in HBA and DES content. The larger the anion, the higher the EB. Dissolving DES strongly affected TPS properties	[28]
U:G 1:1; 2:1 U:S 2:1; U:Gluc:G 1:1:2 U:Fruc:G 1:1:2	potato	thermo- compression	Dissolution tests (polarizable microscopy, DSC), mechanical tests, DMTA, FTIR, TGA, solubility, swelling degrees	U:G as dissolving DES. Higher TS for TPS with DES containing sugars	[29]
U:CC:R 1:1:1	potato + lignin	extrusion	Dissolution test of lignin in DES (microscopy), mechanical tests, DMTA, FTIR, UV-Vis, coloration, solubility, swelling degrees, cone calorimetry	DES as dye and solvent of lignin. High filler content. Fire retardant properties	[93]
U:CC 2:1	potato	casting	Dissolution tests (polarizable microscopy), ARES rheometry of premixtures, mechanical tests, DSC, FTIR, TGA	Different content of DES per starch. Lower thermal stability of TPS/U:CC than TPS/G:CC	[94]
U:G:S 2:2:1	potato + fillers	extrusion	Mechanical tests, DMTA, FTIR, solubility, swelling degrees, cone calorimetry	Comparative of fire-retardant studies with TPS/G	[95]
U:CC 5-2:1 U:B 5-2:1	potato	thermocompression, extrusion	TGA of DESs, mechanical tests, DMTA, FTIR, solubility, swelling degrees	Study of B-type (anhydrous, hydrate, and HCl) influence and urea content on TPS properties	[64]
U:CC 5:1 U:B 5:1	potato + coffee spent grounds	extrusion	Mechanical tests, DMTA, FTIR, solubility, swelling degrees, biodegradability, effect on physico-chemical state of growing plant, total content of nitrogen	Good processability even with high filler content in biocomposite using food waste. Lack of toxic influence on growing plant	[96]
U:CC 5:1 U:B 5:1	wheat (flour) + + coffee spent grounds	extrusion	Mechanical tests, DMTA, FTIR, TGA, solubility, swelling degrees, biodegradability, effect on physico-chemical state of growing plant		[97]
U:CC 2:1	cassava	extrusion	MFI, steady torque, DMTA, XRD, FTIR, TGA, WCA, surface energy	Study of tendency to retrogradation. Comparison with other DESs based on monoethanolamine	[98]
U:CC:G 1:1:1	hydroxypropyl + cellulose and Cu-MOF	casting	Mechanical tests, SEM + elemental mapping, DMTA, FTIR, UV-Vis, TGA, WVTR, WCA, solubility, swelling degrees, biodegradability, antibacterial and cytotoxic properties, molecular dynamics simulations	Comparison with other DESs. MOFs were used as ammonia-responsive indicators. Application of the films as active and smart food tags	[99]
U:CC:G 1:1:1 U:B:G 1:1:1	hydroxypropyl and CuZn-MOF-47	casting			[100]

B—betaine, CC—choline chloride, CAc—choline acetate, CCit—choline citrate, CLa—choline lactate, Fruc—fructose, G—glycerol, Gluc—glucose, MCC—microcrystalline cellulose, MMT—montmorillonite, MOF—metal-organic framework, R—resorcinol, S—sorbitol, U—urea.

The first report describing TPS/U-DES films obtained from gelatinized (cast from aqueous system) corn starch is a study on corn starch-based electrolytes with U:CC and lithium bis(trifluoromethanesulfonyl)imide –LiTFSI [87]. For 20–40% in TPS/LiTFSI, the materials formed films, but for higher content of DES, they became gels, which positively affected ionic conductivity.

The studies on the influence of different types of DESs and pure components (U, G, CC, and choline citrate—CCit) on potato TPS and its derivative, hydroxypropylated oxidized potato starch (HOPS), produced via casting method, including an analysis of their mechanical and barrier properties, were presented in the work of Zdanowicz and Johansson in 2016 [88]. U-DES systems applied in the work are listed in Table 4. Replacement of CC with CCit in DESs led to the formation of films with better mechanical and barrier properties due to the ability of the salt to induce partial crosslinking of the polysaccharide. In the case of behavior of pure components in TPS, the best mechanical and barrier properties were obtained for U. Combined systems with CC led to the creation of films, which were highly flexible and constituted a weaker barrier toward moisture. CC recrystallized in the HOPS after storage, and that process was delayed by the introduction of CC as an eutectic with U or U and G. Moreover, recrystallization appeared faster for non-heated samples. Interestingly, TPS with U exhibited better barrier (water barrier transmission rate—WVTR) toward moisture than TPS/U:CC. HOPS/U began to dissolve under test conditions (RH 70%), whereas HOPS/U:CC exhibited WVTR equal to 756 g/m^2^∙day. U-DES also led to higher water contact angle (WCA) values of starch-based films than G or G-DES.

The work also presented a comparison study of the different ways of introducing the plasticizer (based on CC and U) into TPS: (i) liquid DES (U:CC or choline citrate (CCit):U:G) introduced into starch dispersion before its gelatinization, (ii) DES components introduced separately before gelatinization, and (iii) DES added after starch gelatinization; the best properties were achieved for TPS with DES compounds introduced separately. Even though high water content can disrupt DES structure, new interactions are formed after solvent evaporation. This study, as well as the work of Abbott et al. [83], shows that DES components can exert a synergistic plasticizing effect, leading also to conservation of energy. What is also worth mentioning is the fact that starch-based films with U:CCit:G exhibited excellent barrier toward oxygen (oxygen transmission rate—OTR ca. 1 cm^3^/m^2^∙day).

**Figure 4:**
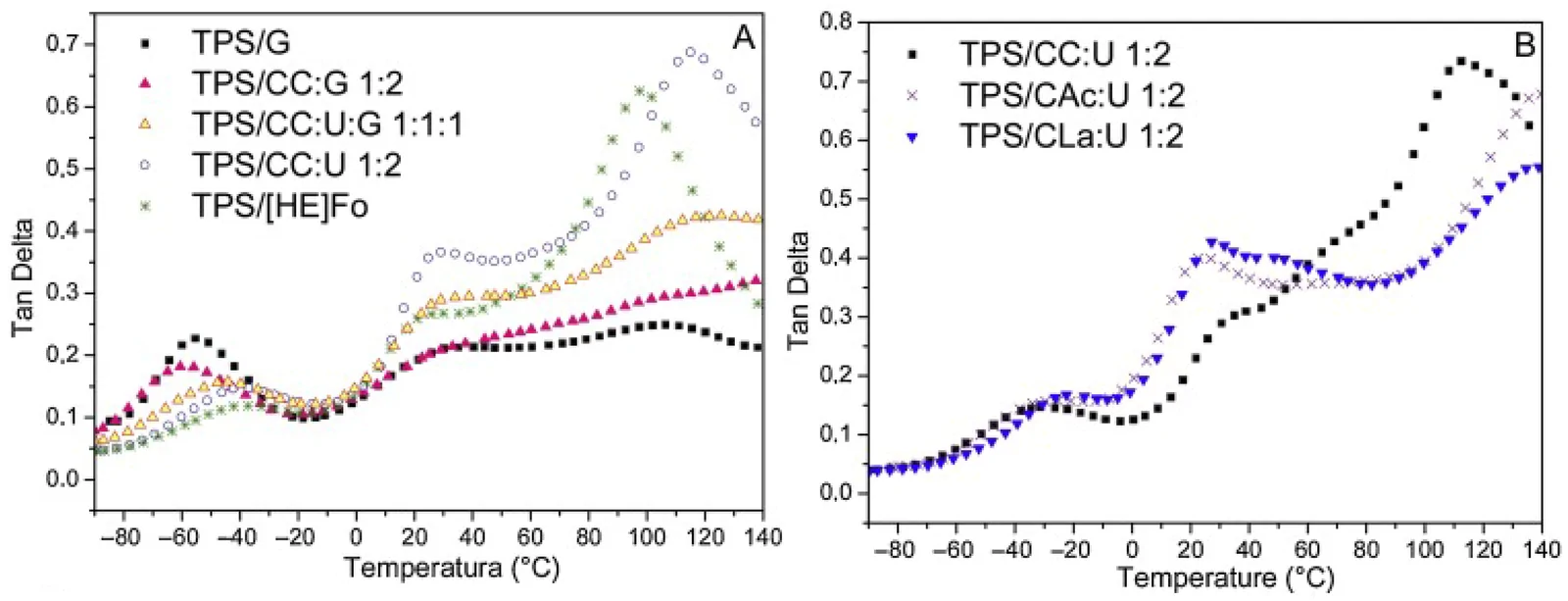
DMTA results (**A**): tan δ for TPS plasticized with different types of treating media; (**B**): tan δ curves for TPS/choline salt:urea films with different types of anion in choline salt. The figure is reproduced with permission from Ref. [28]. Copyright, 2020, Elsevier.

The next work compared several types of different modifiers—DESs, G, and IL—on starch treatment, including dissolution [28]. TPS/DES films obtained via solvent (water)-free thermocompression exhibited similar mechanical properties and processability to TPS/IL. Based on the dissolution test, DESs were divided into two groups: dissolving (IL, U:Choline acetate:U—U: CAc, and U:CC) and non-dissolving (G and G:CC). The division was based on DSC and microscopic analyses. The dissolving systems, including U-DES, led to more amorphous materials, which resulted in higher EB, sorption, and dissolution degrees. These results correspond with earlier studies on the extruded TPS [83,90]. TPS/U-DES was found to be slightly less thermally stable than TPS/G-DES, as in the case of casted films [94], but much higher than in TPS/IL. DMTA analysis revealed a second α-relaxation peak of unfolding polysaccharide chains (Figure 4). This peak was not observed for non-dissolving additives.

Moreover, the viscosity profile during heating was lower for U-DES than starch with G or its DES. This is related to different ways of starch transformation, like the results in work [94], indicating that TPS/U-DES was more easily processable. U-DES dissolved starch, causing the unfolding of polymer chains, starting from external layers of the starch granules, whereas with G (and moisture), the granules swell. The differentiation of these two processes was confirmed by DSC (endothermic peak for gelatinization and exothermic for dissolution) and polarizable microscopy. In Ref. [94], the authors claimed that G:CC also acted as a solvent, but in their microscopy results (DSC was not shown), birefringence and swollen granules could be observed, indicating gelatinization. In the case of the films, the broad, flattened XRD peak for high content of G:CC could be a result of a halo associated with an excess of plasticizer, which in high amounts migrates or “sweats out” from the starch matrix [88,101]. Similar results showing the impact of dissolving DES were presented in another work related to non-choline DESs based on U with polyols (G and/or sorbitol) [29]. Due to DES U:G also acting as a solvent (and is highly polar [63], its use as the plasticizer led to highly amorphous, elastic TPS, whereas DES with sugars that have a lot of -OH groups formed strong bonding with polysaccharides, which led to higher TS, but also—interestingly—to a higher sorption degree (ca. twice higher). It is worth introducing here some works describing TPS with combined systems of plasticizers, where urea was added as one of the co-plasticizers separately. Paluch et al. [102] replaced some amounts of glycerol with U and studied the influence of its presence on, for example, morphology, molecular weight, and thermal properties on extruded potato TPS. For G to U %ratio up to 10:20, TPS was highly amorphous, which can be explained by H-bonding formation between the two additives. Higher U content led to its crystallization in the matrix. According to gel permeation chromatography data, higher U content was associated with a smaller decrease in M_w_, indicating effective plasticization by interactions between hydroxyl groups of the polymer and –NH- from U. In another work describing a similar study on Brabender-kneaded and thermocompressed corn TPS, the increase of U content caused an increase in EB (for G/U 50/50, EB was 30% higher than for G only) [103]. The interactions between U and G were confirmed by NMR analysis in work [104].

Urea with CC at molar ratio 2:1 is liquid at room temperature, but can form eutectic also for higher content of U. Thus, in ref. [64], U with CC or betaine (B) with molar ratio 2:1, 3:1, 4:1, and 5:1 was used to plasticize potato starch by thermocompression. TPS/DES 5:1 premixtures were extruded at 100 or 200 rpm. Before the starch processing, DESs were thermally characterized. The higher U content in DESs with CC and betaine hydrate, the higher Tm, and the lower temperature of decomposition. Since DSC analysis was carried out at temperatures much below 0 °C, glass transition peaks were also observed. In TPS, higher U content increased EB but lowered thermal stability. Comparing processing conditions of the extrusion, increasing the screw rotational speed led to better TPS mechanical properties. Fourier transform infrared spectroscopic analysis revealed that in TPS/U:B 5:1, a small peak at 1772 cm^−1^ assigned to carbamate groups can be observed, indicating that derivatization with U can occur in the presence of betaine. Thermocompressed TPS with DES U:HBA 5:1 maintained their integrity, despite swelling, whereas extruded highly amorphous samples were gelled and defragmented/partially dissolved in water (24 h) [64]. In Ref. [97], starch was successfully extruded with DES U:CC or U:B with molar ratio 1:5 by extrusion in semi-industrial scale. The same process was used for modification of wheat flour, but the films, due to some protein (mainly gluten) content, were more opaque [96]. Aging resistance of cassava TPS depending on different plasticizers was tested in [98]. The studies revealed that TPS with U:CC and monoethanolamine:CC were more resistant to changes, for example, EB, than TPS/G (stored for almost 2 months). An advantage of U-DES is the lower temperature of starch extrusion needed for transformation compared to G or G-DES [20,105], helping to conserve energy.

DES can affect TPS properties by direct treatment. In Tang et al. [62], starch was dissolved in different DESs (Table 2), and regenerated starch was gelatinized. According to simulation calculation results based on density gradients, ternary DES U:B:G exhibited lower H-bonding energy with anhydroglucose units than U:CC:G; thus, the better solubilizing activity led to the disentanglement of the polymer chains. This pretreatment resulted in slightly better mechanical properties and higher moisture permeability and transparency of TPS/U:B:G films than non-pretreated TPS.

U-DESs have been also used as plasticizers for starch-based composites. Zdanowicz and Johansson investigated the impact of different additives: sodium and calcium montmorillonite (MMT), microcrystalline cellulose (MCC), and tannin on the barrier and mechanical properties of thermoplastic potato and hydroxypropylated oxidized starch films with three-component DES CCit:U:G, obtained from casting from aqueous solution [89]. Even though CCit in DES acts as a crosslinking additive [88,101], the results suggest that the presence of MMT can hinder interactions with starch derivative and plasticizer. For TPS, fillers’ presence led to lower EB, WVTR, and higher TS and YM. Tang et al. [99] obtained functional composites based on hydroxypropyl starch (HS), DES CC:U:G (15%) with hardwood cellulose and copper metal organic frameworks (Cu-MOFs) as ammonia-response materials. Addition of the filler led to better mechanical properties, lower WVTR, lower solubility degree, and higher WCA. Similar results were obtained for CuZn-MOF-47 [100]. Density functional theory (DFT)-based computations indicated that the composite exhibited a more complex structure and suggested that cellulose could facilitate reorganization of HS, establishing strong mutual interaction. In Ref. [91], U:CC 2:1 and CC:imidazole (IM) 3:7 were used to obtain extruded TPS composites with urea-modified montmorillonite (UMMT). The films TPS with DES with U were highly elastic, more than with IM with thermal conductivity of ca. 0.24 W/mk that slightly decreased for TPS with UMMT and good processability/thermoformability. Different DESs, including U:CC, were used as plasticizers for starch/wood fiber composites with high content of the filler [92]. DESs, due to their “delignificating” activity, facilitated adhesion between TPS and the filler. A three-component DES made with U:CC and resorcinol (R) was used as the plasticizer for potato starch to obtain its composite with lignin by extrusion [93]. Due to the fact that U can form a colored complex with R, this DES can act as blue dye for the final material. Moreover, the presence of R conferred UV barrier properties on the materials. Since the DES can dissolve lignin, its high content (10 pph per starch) can be added without introducing issues related to thermoprocessing brittleness of the product. Cone calorimetry analysis was performed to investigate the fire behavior of TPS and its composites. This analysis was also conducted for TPS plasticized with chloride-free DES U:G:S [95], indicating that TPS/DES with 10 pph of MMT exhibited much better fire-retardant properties than TPS/G. The works of Zdanowicz et al. show utilization of DES rich in urea for thermoplasticization of starch and wheat flour with high content of spent coffee grounds (20 pph per starch/flour) as functional filler. Physiological state investigations revealed that the thermoformable and biodegradable biocomposites provided a nitrogen source and were nontoxic to a growing model plant [96,97].

## 3. U-DES and Cellulose and Their Derivatives

Cellulose is a polysaccharide consisting of a linear chain of D-glucose units linked with β-(1→4) glucosidic bonding. These strong bonds build a closely packed structure leading to their insolubility in water and in the organic solvents, with some exceptions [106,107]. This type of bonding conditions cellulose’s role as a structural component of the green plants’ cell walls.

In 2005, Laus et al. [108] attempted to dissolve cellulose in DES, but the trial failed; Abbott used U:CC as a medium for O-acylation reaction, but the yield was lower than 5% [25]. Now, it is known that unmodified cellulose is insoluble or barely soluble in DESs, as the presence of DESs is not enough to disrupt strong hydrogen bonding of the biopolymer; therefore, polymer modification facilitates its processing. Cellulose as a thermoplastic is always chemically derivatized—examples include cellulose acetate, cellulose acetate butyrate suitable for extrusion, ethyl cellulose for casting from alcoholic solutions, or water-soluble methyl cellulose, as well as carboxymethyl cellulose (CMC). However, DESs have provided a wide spectrum of processes for cellulose processing, that is, for delignification or nanocellulose preparation [109].

The first report related to cellulose films was the work of Wang et al. [110] in which, at first, cotton linter was dissolved in IL, the gels were pressed and washed; then U:CC with different content of water was added, and the gelled systems were dried. After water evaporation, transparent films were obtained with EB values in the range of 26–35% and TS 4-31 MPa (the parameter decreased with an increase of DES:water ratio). When pure U was added instead of DES, the film was opaque, indicating partial recrystallization of the compound. The work for the first time reported interaction between cellulose and U-DES; however, the presented method, requiring IL utilization, may not be potentially scalable industrially. Cellulosic films with U-DES with different urea content were successively obtained from nanocellulose suspension [111]. DES’ presence positively affected film flatness and uniformity. The CNC/DES systems were slightly more elastic than systems with pure U or polyols and with higher U content in DES. The authors proposed a mechanism of CNC plasticization as ionic attraction between negatively charged sulfate groups of CNC and ammonium cation from CC, as well as formation of new H-bonding with U.

The milestone work for “plastic cellulose-DES” can be the study published by Feng et al. [112], which introduced a novel DES: U:tetramethylammonium hydroxide pentahydrate (TMAH·5H_2_O) 3:1. Different sources of cellulose were dissolved (5 wt%) in DES, and a film was formed by placing the system between glass plates and a coagulation bath. The films’ properties were dependent on the medium used for cellulose regeneration. Films prepared from refined cotton with ethanol exhibited similar properties to PET-G materials. Moreover, the films were characterized by excellent thermal stability. Based on the molecular dynamics simulation, in cellulose exposed to DES, dissociation of polymeric chains caused by strong H-bonding formation occurred, whereas in non-treated cellulose, the bundles maintained their integrity. The role of urea here was the formation of strong H-bonds with cellulose OH groups.

In Ref. [113], U:CC was added to hydrophobic acetate, whereas the work presented system with sodium acetate U:(NaAc) and CMC as a matrix, but DES acted more as a functional additive than a plasticizer.

## 4. Other Non-Starch Polysaccharides

**Figure 5:**
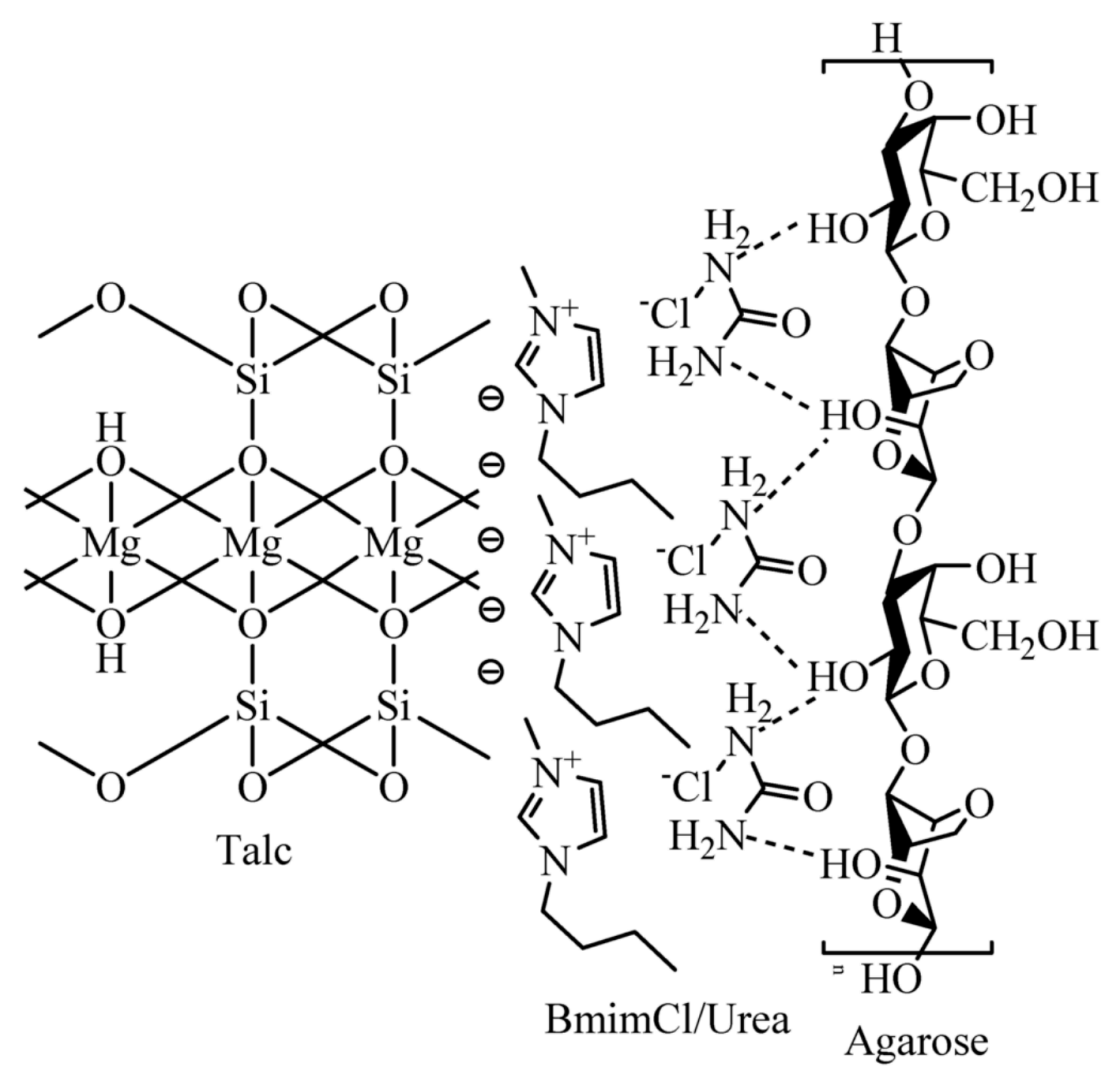
Proposed model of mutual interactions between talc, IL ([BMIM]Cl/urea, and agarose) [115].

Shamsuri and Daik prepared highly transparent agarose films with 30% of U:CC by casting from aqueous solution [114]. Mechanical tests (results: lower TS, higher EB, lower crystallinity degree, and lower T_g_) confirmed the plasticizing effect of the DES on agarose. For 70% DES content, the weakening of mechanical properties was observed due to phase separation. Another work of the authors presented some studies on composite agarose films with talc (4 wt%) and IL: [BMIM]Cl combined with urea. Due to the high cost of ILs, the introduction of U can lower the price of the final material. In opposition to the previous study, an increase in mixture content led to higher T_g_. This phenomenon was explained by interfacial adhesion between the filler and the agarose/DES matrix. DSC and SEM results revealed that U:IL acted as a coupling agent [115]. The proposed model of talc–U/IL–agarose interactions is presented below (Figure 5).

The other polysaccharide plasticized with U:CC was agar [116]. A three-step method of film formation was proposed: the polysaccharide was dissolved (2–6%) in DESs, thermocompressed, and washed in ethanol. Agar pretreated with U:CC exhibited better film-forming ability than in G:CC. In the next work [117], film’s preparation by thermocompression was adopted from work [83], and additionally, the influence of pressing conditions on agar films was investigated. Optimal conditions for mechanical properties were 140 °C, 20 min, and 176 kN; however, noticeable changes in color (darker and brownish) were noted. Pure U as the plasticizer behaved as in the other polysaccharide matrices, that is, corn starch [83], HOPS [88], or CNC [111], and recrystallized. In the work [31], U:CC/agar gels (excess of DES in the system) find application as proteinaceous-based varnishes from paintings.

U-DES has also found applications for ionic polysaccharides. In Ref. [118], U:CC was used as an additive for chitosan (Ch) films, and its influence on proton conduction was studied. A blend of Ch with CMC with a weight ratio 1:1 exhibited the highest conductivity, that is, facilitated by DES addition (there is no information about the content). Interestingly, even though DES is highly hydrophilic, the films modified with DESs exhibited lower water uptake and inhibited film disruption in water. The authors indicated that this behavior could have resulted from the occurrence of ionic interactions between the polysaccharides and DES components. Ashaf et al. [119] presented a study of the influence of CC-based DESs with U, ethylene glycol, and lactic acid (La) on pullulan/carboxymethyl Ch/films with zein-nisin addition (5, 10, and 15%). However, in this case, the use of CC:La was advantageous over U:CC when comparing mechanical and barrier properties. Blend films made with chitosan and gelatin (1:1 weight ratio) were modified with U:CC with extract from *Dalbergia pinnata* Prain to obtain active packaging. First, DES was used for Prain extraction to enrich the medium with polyphenols (DPF); then DES/DPF was added to the biopolymer formulation with different contents. The film without additive was very brittle, whereas for the higher studied content of DES/DPF, EB reached ca. 90% with drop in TS, indicating plasticizing activity. DPF presence gave the films some antibacterial and antioxidative properties. Authors indicated that DES improved the stability of the flavonoid extract in the matrix, but there is no comparative study without DES in their article [120].

Kowalonek et al. [121] used different DESs for extraction of active compounds from lemon balm and chokeberry pomace. Extract-in-DES was introduced into alginate solution; then the system was cast to evaporation. DESs containing urea were prepared with betaine and glucose (Gluc). Both types of DES and extract affected final film properties; for example, alginate film with U:Gluc was more flexible than with U:B. These results are interesting and opposite to results presented in [29], where starch modified with U:glucose eutectics exhibited higher TS than TPS/U:G. This can be an effect of extract presence and casting method of film preparation. Moreover, films with U:Gluc DES were more antioxidative, meaning that they were able to extract more active compounds from the plant sources. The application of DESs as plasticizers for non-starch polysaccharides are listed in Table 5.

## 5. Urea-DESs as Proteins Plasticizers

Proteins are biological macromolecules, and as polymers, they are quite unique. The molecular weight is typically in the range of 33–55 kDa, but a macromolecule is constituted by different amino acids (determining properties such as electrical charge or solubility), as opposed to polysaccharides that possess a repetitive sequence of sugars.

Deep eutectic solvents can be used as solvents, extraction media, or stabilizers of the molecular structure of proteins [123,124], which is especially important for enzymes [125,126]. DES activity toward proteins can be highly affected by water presence [127,128,129]. Urea is a denaturant for proteins but, interestingly, combined with other compounds, can act as an enzyme stabilizer, affecting their reactivity. Monhemi et al. [42] showed that U:CC stabilized lipase sensitive to pure U. The lack of denaturizing activity of urea was caused by the formation of H-bonding of U with CC, which limited U diffusion into enzyme molecules. Even when U and CC were applied separately, before eutectic formation, lipase preserved its initial structure in the combined system [130]. Some researchers tried to find out if plasticization of proteins with DESs could be challenging. The first report with protein and DES as plasticizer was in [84], but zein was used in a blend with starch in quite low content (10 wt%). Five years later, zein with IL and DESs (30%) was extruded in a micro-compounder; unfortunately, the mechanical properties of zein/U:CC 2:1 were far from satisfactory. TS was 2 MPa, and EB was equal to 3%; much better results were obtained for ILs [90] DE G:CC [131] and CC:ethylene poly(ethylene glycol) [132]. Yang et al. [120] used U:CC for the extraction of *Dalbergia pinnata* L. Prain and preparation of active films from the blend of gelatin/chitosan by casting solution (this example was also presented in the Section 4, Table 5). The addition of extract-in-DES led to transparent, flexible films. With the increase of the additive, WCA and water permeability increased, and solubility in water decreased. The films were used as fruit packaging, and the source of components should be taken into consideration for potential implementation. Utilization of gelatin needs proper labeling due to ethical (not suitable for vegans) or religious reasons. Eutectic mixture U:NaAc was an effective modifier for gelatin-based materials [32,133]. In [133] first, the protein was dissolved in DES:water, and then the gel was mixed with poly(vinyl alcohol)—PVA solution. As a crosslinking agent, N-(3-dimethylaminopropyl)-N’-ethylcarbodiimide hydrochloride was used. DES-pretreatment of gelatin destroyed intermolecular bonding that facilitated film formation. With increase of DES/gelatin in the blend, WCA, water permeability, and EB increased, and thermal stability was maintained. U:CC 2:1 was used to dissolve cow horn (1:20 mass ratio), and the whole system was added to soy protein isolate in different amounts and then thermocompressed. The DES/horn mixture acted as the plasticizer, leading to high EB values, exceeding 80% for 75% content of the combined plasticizer. Pure thermocompressed SPI was used as a control, so the results do not show the effect of DES itself on the SPI. Additionally, the authors presented environmental assessments showing a minimal environmental impact of the main relative contributors of the environmental load: procedures included DES preparation and soybean production [134]. This is the first work related to DES additive for biopolymers or bioplastics, indicating the growing importance of LCA (life cycle assessment). So far, LCA for DES-biopolymer has been determined, mostly focusing on DESs as pretreatment media; however, due to limited collected data, the review [135] shows more potential environmental impact that needs to be studied.

Urea can facilitate plasticizing gluten or rice protein when it is added to systems with polyols by solvent-free methods. Türe et al. [136] added U into wheat gluten (WG)/G premixture to obtain thermoplasticized WG films, with EB reaching almost 90% for 30 pph per WG/G. In Ref. [137], rice protein (RP) with U and different polyols was processed by injection molding. RP film with 35 pph of U and 15 pph G exhibited good mechanical properties (EB 40%), and DSC analysis showed the shift of melting point assigned to U from ca. 130 to 90 °C, as well as a noticeable decrease of ΔH, which indicates mutual interactions of both plasticizers and the formation of eutectic forms, leading to a synergistic effect on plasticization, similarly as in starch [83].

## 6. Summary

**Figure 6:**
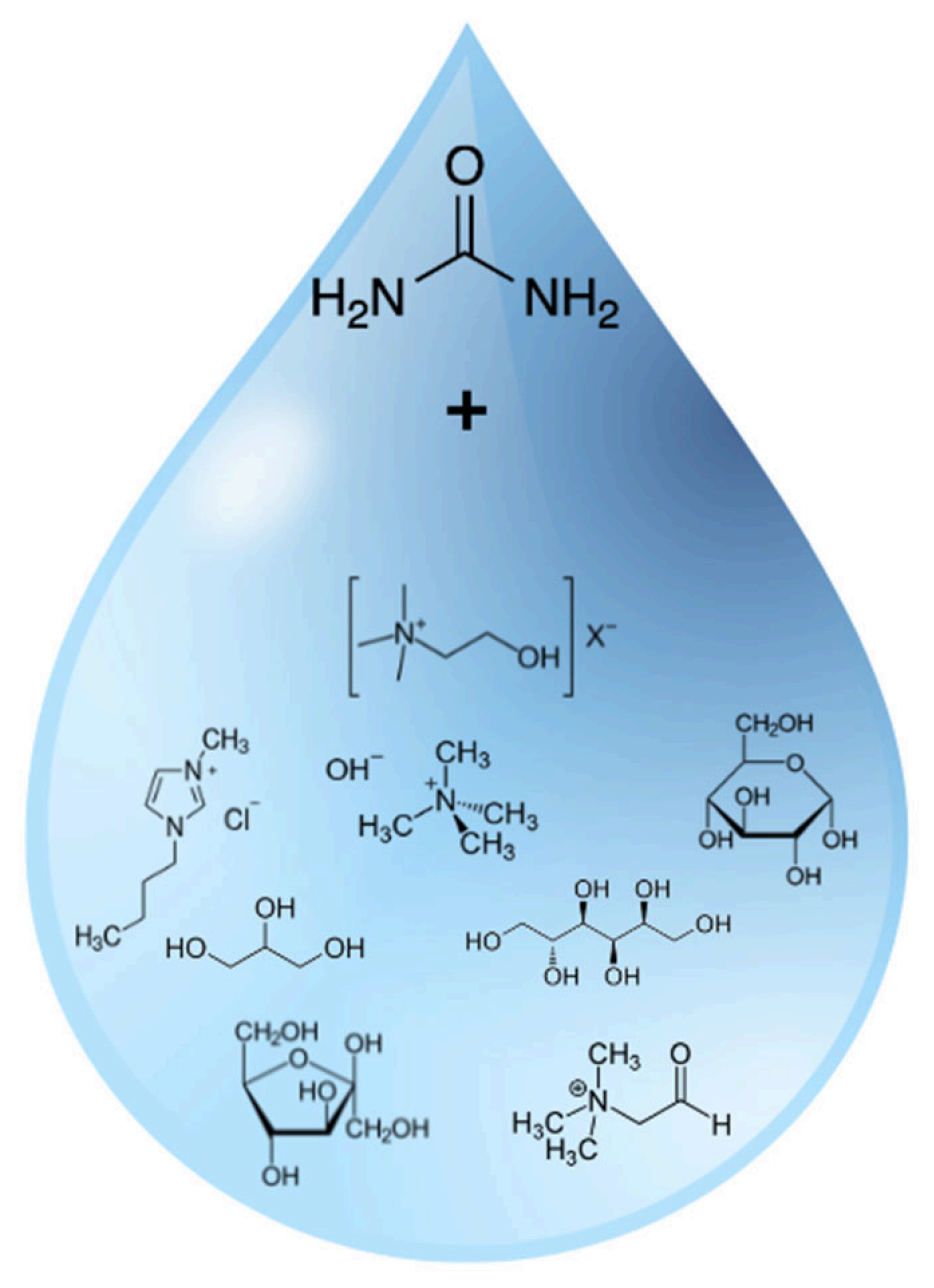
Compounds form eutectic mixtures with urea, which was used for polysaccharides and proteins plasticization.

The most popular and studied DES used in the area of biopolymers is the mixture of urea and choline chloride, but eutectics with other choline salts, polyols, or sugars have been studied (Figure 6).

**Figure 7:**
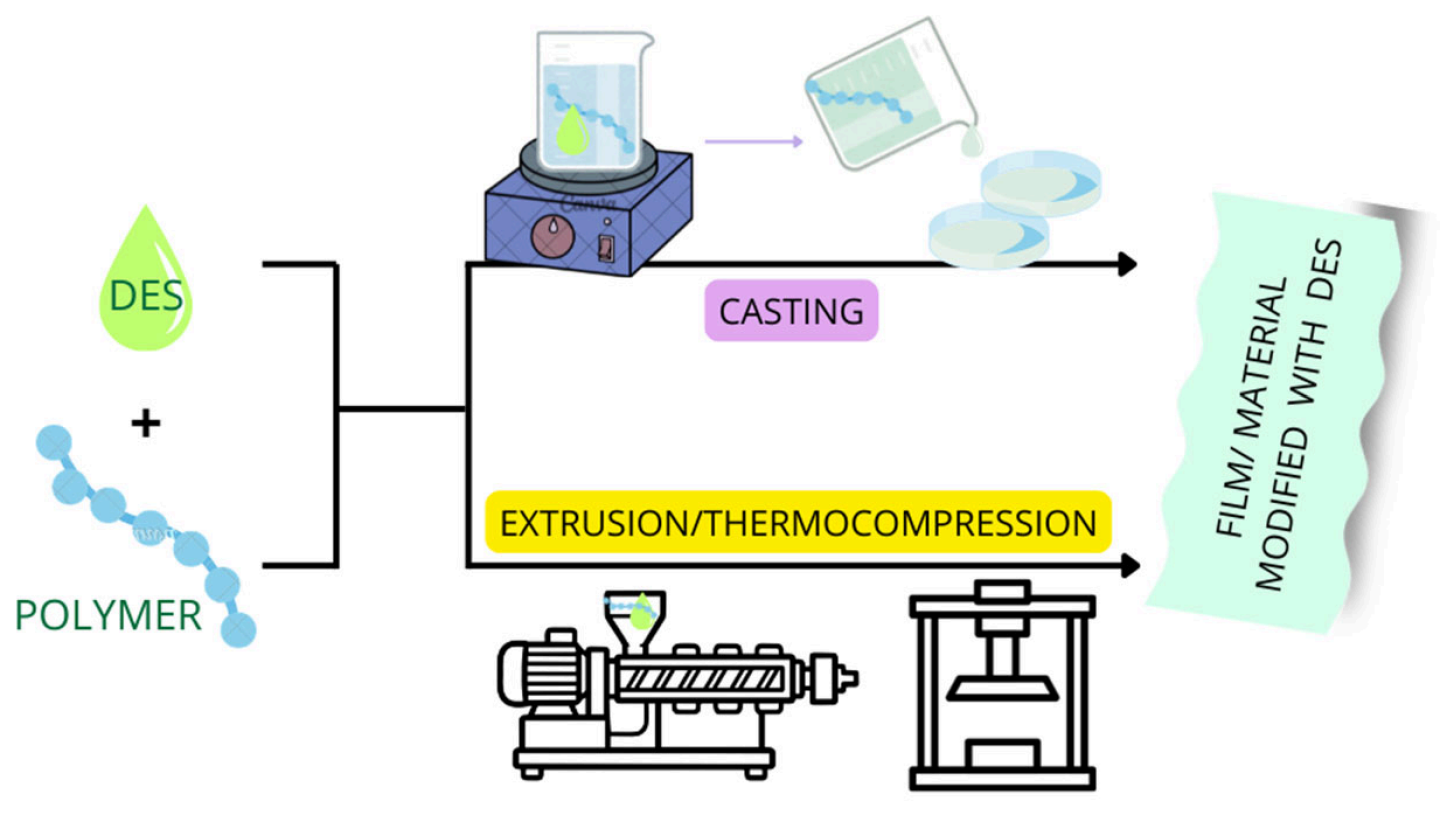
Methods of plasticization of biopolymers with U-DESs.

The preparation of plasticized biopolymer materials uses the casting method from aqueous solutions/dispersions and gels and via solvent-free techniques involving thermocompression and extrusion (Figure 7). Extrusion as a continuous method that does not require solvent and evaporation process has huge potential for industrial scaling.

U-DESs acted similarly to ionic liquids toward starch, being cheaper, more available, and more thermally stable than ILs.

U-DESs as plasticizers for biomacromolecules can be summarized in a few points:

(a) Starch is the most common biopolymer used for treatment with U-DES.
(b) U-DESs acted as primary plasticizers, dissolving all fractions of the polysaccharide (similarly to molecular ionic liquids), leading to highly amorphous extruded materials with high EB [28,29,84,90,94]. Low TS for polymer/U-DES can be improved by alternation of DES composition, for example, application of ternary DES [28,29,88,95,99,100] or use of compounds forming more H-bonding [88,95], addition of fillers [89,91,92,96,97,99,100,115] or partial crosslinking [88,91].
(c) Materials with U-DESs exhibited a less hydrophilic (lower WCA) surface than with conventional plasticizer or G-DESs [28,88,91,92,99,100].
(d) Physicochemical properties of final plasticized materials are dependent on processing method, water/moisture content, and DES components’ type and ratio.
(e) TPS/U-DES are less thermally stable than TPS/G and TPS/G-DES but exhibit higher thermal stability than TPS/IL [28,94]. The higher U content, the lower thermal stability of polysaccharide material [64,110,120].
(f) U-DESs can act as compatybilizing/coupling plasticizers [84,90,91,92,93,96,97,99,115].
(g) High content of the filler can be introduced (more than 10 pph per polymer) into materials plasticized with U-DESs [92,95,96,97].
(h) DES components introduced separately exhibited synergistic activity, affecting high plasticization degree of polysaccharides and proteins [83,84,88,102,117].
(i) U-DESs exhibit additional functionality, for example, crosslinking [88,89], dyeing [93], UV barrier [93], conductivity [113,118,122], fire retardancy [93,95], nitrogen source [96,97], and extraction media/carrier of active compounds [93,96,120,121].
(j) Antimicrobial and/or antioxidative properties of the materials can be obtained by incorporation of active additives, for example, extracts and particles [99,100,120,121].

Due to the functionality of U-DESs, modified biopolymers can be used as biodegradable food packaging, as conductive or agricultural materials.

## 7. Future Directions

Generally, polysaccharides, due to their less complex structure than proteins, are more compatible with U-DESs; however, only a few U-DESs have been studied so far. Thus, there is a broad unexplored area for designing U-DESs for different proteins. DESs as tailorable media could be designed for specific proteins. Moreover, DESs are widely used as media for the extraction of biomacromolecules and active compounds; hence, combined systems (e.g., extractant as plasticizer + extracted substances) could be applied to different materials, not only for pure matrices but also for blends and composites, giving additional functionality. As some works indicated, DES-modified films can be applied as food packaging/coating; therefore, migration of the additives needs to be studied. This issue is also related to toxicity and cytotoxicity, which have been reported in only a few works so far, but in these studies, the films were modified with active particles. Only studies of the influence of U-DES-rich materials as agricultural plastics on growing plants were conducted [96,97]. The type of interaction in DESs molecules was investigated by molecular simulations, but DES–polymer matrix interaction has not been studied deeply, except for Tang et al. [99,100], so there is a wide area for future research. LCA was performed for DESs, but more as reaction or extraction media, indicating that their sustainability is disputable, for example, the source of the mixture components, despite generally indicating the green character of the eutectics. However, many comparisons refer to molecular ionic liquids. Glycerol, the most popular plasticizer for biomacromolecules, is obtained from natural sources; thus, the LCA comparing different plasticizers, including DESs, needs to be provided to look at DESs in a wider context. The influence of water presence on biopolymer transformation, like dissolution, is known, but still, there is a lack of comparative studies on water presence/content on plasticization.

## Figures and Tables

**Table 1:** Freezing point of DESs made with choline chloride with urea and its derivatives in a molar ratio 1:2 [11].

Amide	T_f_ (°C) of Amide	T_f_ (°C) of DES
Urea	134	12
1-methylurea	93	29
1,3-dimethyl urea	102	70
1,1-dimethyl urea	180	149
Thiourea	175	69

**Table 2:** Results of toxicological tests and degradation in water of U:CC compared to mixtures of individual components and CC-DES with polyols [58].

Medium	MIC *A. niger* [mg/mL]	Zone Inhibition *A. niger* [mm]	Aquatic LC_50_ [mg/L]	Degradation for 2 Weeks [%]
U	126	n.m.	n.m.	n.m.
CC	628	n.m.	12,478	n.m.
U:CC 2:1	138	14 for 200 mg	5310	85
U + CC indiv	n.m.	n.m.	4441	n.m.
G:CC 2:1	550	4 for 300 mg	8754	91
EG:CC 2:1	325	10 for 300 mg	10,076	77

n.m.—not measured.

**Table 4:** Mechanical and barrier properties of native potato thermoplastic starch (TPS) and hydroxypropylated oxidized potato starch (HOPS) plasticized with U-DES with the comparison with pure glycerol (G) and U [88].

Plasticizer	EB [%]	TS [MPa]	YM [MPa]	WVTR_RH70%_	WCA [°]	MS [%]
TPS						
without	9 ± 1	46 ± 10	1190 ± 50	344 ± 45	70 ± 5	8.1 ± 0.6
G	95 ± 15	5 ± 1	120 ± 11	407 ± 23	54 ± 6	8.3 ± 0.2
U	26 ± 8	12 ± 3	496 ± 108	330 ± 25	40 ± 7	6.3 ± 0.3
CC:U 1:2	74 ± 16	3 ± 0	126 ± 13	675 ± 41	55 ± 5	8.9 ± 0.3
CC:U:G 1:1:1	71 ± 22	4 ± 1	133 ± 37	549 ± 21	62 ± 7	8.1 ± 0.4
CCit:U 1:2	13 ± 7	17 ± 7	518 ± 26	159 ± 26	41 ± 5	5.6 ± 1.3
CCit:U:G 1:2:2	30 ± 8	10 ± 3	410 ± 50	211 ± 21	73 ± 10	6.5 ± 0.5
CC:U:S 1:1:1	20 ± 15	11 ± 5	124 ± 18	250 ± 51	62 ± 5	7.2 ± 0.0
HOPS						
without	too brittle					
G	76 ± 11	1 ± 0	27 ± 3	564 ± 51	45 ± 4	9.3 ± 0.0
U	80 ± 12	4 ± 1	151 ± 46	n.m	49 ± 7	7.6 ± 0.1
CC:U 1:2	211 ± n.d.	1 ± 0	30 ± 10	756 ± 14	n.m.	n.m
CC:U:G 1:1:1	crystallized					
CCit:U 1:2	32 ± 8	6 ± 1	320 ± 60	n.m.	53 ± 4	5.4 ± 0.3
CCit:U:G 1:2:2	55 ± 7	5 ± 1	148 ± 15	352 ± 37	54 ± 3	6.7 ± 0.3
CC:U:S 1:1:1	87 ± 22	2 ± 1	116 ± 23	464 ± 7	n.m	8.3 ± 0.3

CC—choline chloride, CCit—choline citrate, G—glycerol, U—urea; n.d—not determined; n.m—not measured.

**Table 5:** Non-starch polysaccharides modified with U-DES.

Polysaccharide	U-DES	Method of Film Preparation	Methods	Remarks	Ref
Regenerated cellulose	U:CC 2:1	casting	Mechanical tests, FTIR, XRD, AFM, SEM	First, cellulose was regenerated in IL. Study of the influence of DES content in water	[110]
	U:TMAH (×5H_2_O) 3:1	gel spreading	Molecular dynamic simulations for regenerated cellulose; mechanical tests, FTIR, XRD, TGA, WCA	The films were formed between glass plates. High thermal stability and mechanical properties similar to PET-G	[112]
CNC	U:CC 1:2; 1:1; 2:1; 4:1	casting	Mechanical tests, FTIR, polarizable microscopy, XRD, AFM, SEM	Films exhibited a smooth surface; the best performance was for 2:1	[111]
Agar	U:CC 2:1	thermocompression	Mechanical tests, SEM, sorption isotherms, WVP, WCA	Agar was dissolved in DES before compressing	[116]
	U:CC 2:1	thermocompression	Mechanical tests, SEM, sorption isotherms, WVP, WCA, coloration, and opacity	CC and U added separately, comparison with pure U, study of pressing conditions influence	[117]
Agarose	U:CC 2:1	casting	Mechanical tests, FTIR, UV-Vis, DSC, TGA	Study of DES different content	[112]
	U:[BMIM]Cl 1:1 + talc	casting	Mechanical tests, FTIR, Raman spectroscopy, DSC, TGA	DES as coupling agent between matrix and talc	[115]
Chitosan	U:CC 2:1	casting	Conductivity, sorption, proton/ion exchange capacity, FTIR, SEM	DESs led to better integrity of soaked films, increased proton conductivity	[122]
Chitosan/ carboxymethyl cellulose	U:CC 2:1	casting	Conductivity, sorption, proton/ion exchange capacity, FTIR, SEM, TGA, DSC		[118]
Chitosan/ gelatin	U:CC 2:1 + extract	casting	Mechanical tests, TGA, OP, SEM, XRD, FTIR, XPS, UV-Vis, WVP, WCA, coloration, biodegradation, anti-pathogenicity, cytotoxicity	DES/extract system acted as active plasticizer. The films were used as fruit packaging	[120]
Pullulan/ carboxymethyl chitosan	U:CC 2:1	casting	Mechanical tests, WVP, OP, SEM, XRD, FTIR, biodegradation, anti-pathogenicity	Films with U:CC exhibited worse mechanical and barrier properties than for CC:La; tested as food coating	[119]
Alginate	U: Gluc 1:1 U:B 1:1 + extracts	casting	Mechanical tests, coloration, antioxidative properties	DES as simultaneous extractant and plasticizer for active food packaging films	[121]

B—betaine, [BMIM]Cl—1-buyl-3-methylimidazolium chloride, CNC—cellulose nanocrystals, CC—choline chloride, G—glycerol, Gluc—glucose, TMAH—tetramethylammonium hydroxide, U—urea.

## Data Availability

No new data were created or analyzed in this study. Data sharing is not applicable to this article.

## Abbreviations

The following abbreviations are used in this manuscript:

AFM	Atomic force microscopy
[AMIM]Cl	1-Allyl-3-methylimidazolium chloride
B	Betaine
[BMIM]Cl	1-Butyl-3-methylimidazolium chloride
CAc	Choline acetate
CC	Choline chloride
CCit	Choline citrate
Ch	Chitosan
CLa	Choline lactate
CMC	Carboxymethyl cellulose
CNC	Cellulose nanocrystals
DES	Deep eutectic solvent
DFT	Density functional theory
DMTA	Dynamic mechanical thermal analysis
DSC	Differential scanning calorymetry
EB	Elongation at break
Fruc	Fructose
FTIR	Fourier transform infrared spectroscopy
G	Glycerol
Gluc	Glucose
HBA	Hydrogen bond acceptor
HBD	Hydrogen bond donor
HOPS	Hydroxypropyl oxidized potato starch
La	Lactic acid
LCA	Life cycle assessment
MCC	Microcrystalline cellulose
MFI	Melt flow index
MMT	Montmorillonite
NaAc	Sodium acetate
OP	Oxygen permeability
OTR	Oxygen transmission rate
R	Resorcinol
RP	Rice protein
S	Sorbitol
SEM	Scanning electron microscopy
SPI	Soy protein isolate
TGA	Thermal gravimetric analysis
TMAH	Tetramethylammonium hydroxide
TPS	Thermoplastic starch
TS	Tensile strength
U	Urea
UMMT	Urea-modified montmorillonite
WCA	Water contact angle
WG	Wheat gluten
WVP	Water vapor permeability
WVTR	Water vapor transmission rate
XPS	X-ray photoelectron spectroscopy
XRD	X-ray diffractometry
YM	Young’s modulus

## References

[1] Urea (2021). I Was the Key to a Pioneering Chemical Discovery. What Molecule Am I?. https://www.acs.org/molecule-of-the-week/archive/u/urea.html.

[2] Urea Market Dynamics—Global Trends, Prices, and Trade Flow Insights. https://www.greengubregroup.com/blogs/urea-market-dynamics-global-trends-prices-and-trade-flow-insights.

[3] (2025). Production of Urea Worldwide from 2009 to 2024. https://www.statista.com/statistics/1287028/global-urea-production.

[4] Swify S., Mažeika R., Baltrusaitis J., Drapanauskaitė D., Barčauskaitė K. (2024). Review: Modified Urea Fertilizers and Their Effects on Improving Nitrogen Use Efficiency (NUE). Sustainability.

[5] Kurzer F., Sanderson P.M. (1956). Urea in the History of Organic Chemistry Isolation from Natural Sources. J. Chem. Edu..

[6] Ramberg P. (2000). The Death of Vitalism and the Birth of Organic Chemistry: Wohler’s Urea Synthesis and the Disciplinary Identity of Organic Chemistry. Ambix.

[7] Meessen J. (2014). Urea Synthesis. Chem. Ing. Tech..

[8] Volz N., Clayden J. (2011). The Urea Renaissance. Angew. Chem. Int. Ed..

[9] Imperato G., Eibler E., Niedermaier J., König B. (2005). Low-Melting Sugar-Urea-Salt Mixtures as Solvents for Diels-Alder Reactions. Chem. Commun..

[10] Liang H., Li H., Wang Z., Wu F., Chen L., Huang X. (2001). New Binary Room-Temperature Molten Salt Electrolyte Based on Urea and LiTFSI. J. Phys. Chem. B.

[11] Abbott A.P., Capper G., Davies D.L., Rasheed R.K., Tambyrajah V. (2003). Novel Solvent Properties of Choline Chloride/Urea Mixtures. Chem. Commun..

[12] Ravishankar K., Muthusamy S., Durai S.K., Murugan G., Koushik A.V.V., Thirumal N., Bhaskar S.N., Jaisankar S.N. (2024). Microwave-Assisted Carbamation and One-Pot Phosphorylation-Carbamation of Starch Using Molten Urea as a Reactive Solvent. ACS Sustain. Resour. Manag..

[13] Mita H., Nomoto S., Terasaki M., Shimoyama A., Yamamoto Y. (2005). Prebiotic Formation of Polyamino Acids in Molten Urea. Int. J. Astrobiol..

[14] Sha M., Zhang S., Ju B. (2025). Starch-Based Flocculants Prepared by a Reactive Deep Eutectic Solvent Based on Solid-Phase Method. Int. J. Biol. Macromol..

[15] Tomas V.K., Belyanin V.A. (1972). The Improved Wear Resistance of Gray Irons After Low-Temperature Liquid Cyaniding. Met. Sci. Heat Treat..

[16] Reynaga-Navarro W., Bozzo L., Wijffels R.H., Eppink M., Kazbar A. (2026). Characterisation of Alginate Extracted from Saccharina Latissima with Deep Eutectic Solvents. Food Hydrocoll..

[17] Wada T., Kimura F., Yamamoto R. (1983). Studies on Salt Hydrate for Latent Heat Storage, II. Eutectic Mixture of Pseudo-Binary System CH_3_CO_2_Na·3H_2_O–CO(NH_2_)_2_. Bull. Chem. Soc. Jpn..

[18] Hansen B.B., Spittle S., Chen B., Poe D., Zhang Y., Klein J.M., Horton A., Adhikari L., Zelovich T., Doherty B.W. (2021). Deep Eutectic Solvents: A Review of Fundamentals and Applications. Chem. Rev..

[19] Francisco M., van den Bruinhorst A., Kroon M.C. (2012). New Natural and Renewable Low Transition Temperature Mixtures (LTTMs): Screening as Solvents for Lignocellulosic Biomass Processing. Green Chem..

[20] Zdanowicz M., Staciwa P., Spychaj T. (2019). Low Transition Temperature Mixtures (LTTM) Containing Sugars as Potato Starch Plasticizers. Starch-Stärke.

[21] Nejrotti S., Antenucci A., Pontremoli C., Gontrani L., Barbero N., Carbone M., Bonomo M. (2022). Critical Assessment of the Sustainability of Deep Eutectic Solvents: A Case Study on Six Choline Chloride-Based Mixtures. ACS Omega.

[22] Zdanowicz M., Paszkiewicz S., El Fray M. (2025). Polyesters and Deep Eutectic Solvents: From Synthesis through Modification to Depolymerization. Prog. Polym. Sci..

[23] Zdanowicz M., Wilpiszewska K., Spychaj T. (2018). Deep Eutectic Solvents for Polysaccharides Processing. A Review. Carbohydr. Polym..

[24] Lončarić M., Jakobek L., Molnar M. (2021). Deep Eutectic Solvents in the Production of Biopolymer-Based Materials. Croat. Chem. Acta.

[25] Abbott A.P., Bell T.J., Handa S., Stoddart B. (2005). O-Acetylation of Cellulose and Monosaccharides Using a Zinc Based Ionic Liquid. Green Chem..

[26] Wang Q., Yao X., Tang S., Lu X., Zhang X., Zhang S. (2012). Urea as an Efficient and Reusable Catalyst for the Glycolysis of Poly(Ethylene Terephthalate) Wastes and the Role of Hydrogen Bond in this Process. Green Chem..

[27] Wang Q., Yao X., Geng Y., Zhou Q., Lu X., Zhang S. (2015). Deep Eutectic Solvents as Highly Active Catalysts for the Fast and Mild Glycolysis of Poly(Ethylene Terephthalate)(PET). Green Chem..

[28] Zdanowicz M. (2020). Starch Treatment with Deep Eutectic Solvents, Ionic Liquids and Glycerol. A Comparative Study. Carbohydr. Polym..

[29] Zdanowicz M. (2021). Deep Eutectic Solvents Based on Urea, Polyols and Sugars for Starch Treatment. Int. J. Biol. Macromol..

[30] Depoorter J., Mourlevat A., Sudre G., Morfin I., Prasad K., Serghei A., Bernard J., Fleury E., Charlot A. (2019). Fully Biosourced Materials from Combination of Choline Chloride-Based Deep Eutectic Solvents and Guar Gum. ACS Sustain. Chem. Eng..

[31] Jia Y., Sciutto G., Botteon A., Conti C., Focarete M.L., Gualandi C., Samorì C., Prati S., Mazzeo R. (2021). Deep Eutectic Solvent and Agar: A New Green Gel to Remove Proteinaceous-Based Varnishes from Paintings. J. Cult. Herit..

[32] Cui Y., Zhu Y., Dai R., Shan Z., Yi J., Chen H. (2021). The Solubility and Interactions of Gelatin in “Water-in-Sodium Acetate Trihydrate/Urea-DES” System. Colloids Surf. A Physicochem. Eng. Asp..

[33] Jiang Z., Yuan J., Wang P., Fan X., Xu J., Wang Q., Zhang L. (2018). Dissolution and Regeneration of Wool Keratin in the Deep Eutectic Solvent of Choline Chloride-Urea. Int. J. Biol. Macromol..

[34] Turan O., Isci A., Yılmaz M.S., Tolun A., Sakiyan O. (2024). Microwave-Assisted Extraction of Pectin from Orange Peel Using Deep Eutectic Solvents. Sustain. Chem. Pharm..

[35] Zdanowicz M., Spychaj T., Maka H. (2016). Imidazole-Based Deep Eutectic Solvents for Starch Dissolution and Plasticization. Carbohydr. Polym..

[36] Vorobyova V., Skiba M., Vinnichuk K., Linyucheva O., Vasyliev G. (2025). Influence of Hydrogen Bond Donor Compound Type on the Properties of Betaine Based Deep Eutectic Solvents: A Computational and Experimental Approach. J. Mol. Struct..

[37] Abd-El-Aly I.S., Mekkawy S.A., Hebishy A.M.S., Mahmoud A.R., Abdel Moneim A.E., Abdelfattah M.S. (2026). Tailored Ternary Deep Eutectic Solvents Based on Choline Chloride, Urea, and Oxalic Acid: Preparation, Characterization and Application for Polysaccharide Extraction. J. Solut. Chem..

[38] Sun H., Li Y., Wu X., Li G. (2013). Theoretical Study on the Structures and Properties of Mixtures of Urea and Choline Chloride. J. Mol. Model..

[39] Ashworth C.R., Matthews R.P., Welton T., Hunt P.A. (2016). Doubly Ionic Hydrogen Bond Interactions within the Choline Chloride-Urea Deep Eutectic Solvent. Phys. Chem. Chem. Phys..

[40] Javanmard T., Jabbari M., Nabavi-Amri S.A., Jabbari A. (2026). Novel Sustainable Choline Chloride-Free Deep Eutectic Solvents: Preparation and Physicochemical Evaluation. Mater. Res. Bull..

[41] Hammond O.S., Bowron D.T., Edler K.J. (2016). Liquid Structure of the Choline Chloride-Urea Deep Eutectic Solvent (Reline) from Neutron Diffraction and Atomistic Modelling. Green Chem..

[42] Monhemi H., Housaindokht M.R., Moosavi-Movahedi A.A., Bozorgmehr M.R. (2014). How a Protein Can Remain Stable in a Solvent with High Content of Urea: Insights from Molecular Dynamics Simulation of Candida Antarctica Lipase B in Urea: Choline Chloride Deep Eutectic Solvent. Phys. Chem. Chem. Phys..

[43] Fetisov E.O., Harwood D.B., Kuo I.F.W., Warrag S.E.E., Kroon M.C., Peters C.J., Siepmann J.I. (2018). First-Principles Molecular Dynamics Study of a Deep Eutectic Solvent: Choline Chloride/Urea and Its Mixture with Water. J. Phys. Chem. B.

[44] Zhekenov T., Toksanbayev N., Kazakbayeva Z., Shah D., Mjalli F.S. (2017). Formation of Type III Deep Eutectic Solvents and Effect of Water on Their Intermolecular Interactions. Fluid Phase Equilib..

[45] Sapir L., Harries D. (2020). Restructuring a Deep Eutectic Solvent by Water: The Nanostructure of Hydrated Choline Chloride/Urea. J. Chem. Theory Comput..

[46] Nava-Ocampo M.F., Al Fuhaid L., Santana A., Bucs S.S., Verpoorte R., Hae Choi Y., Witkamp G.J., Vrouwenvelder J.S., Farinha A.S.F. (2021). Structural Properties and Stability of the Betaine-Urea Natural Deep Eutectic Solvent. J. Mol. Liq..

[47] Di Pietro M.E., Tortora M., Bottari C., Colombo Dugoni G., Pivato R.V., Rossi B., Paolantoni M., Mele A. (2021). In Competition for Water: Hydrated Choline Chloride:Urea vs Choline Acetate:Urea Deep Eutectic Solvents. ACS Sustain. Chem. Eng..

[48] Khodabandeh R., Zolghadr A.R. (2025). Structural and Interfacial Behavior of Choline Chloride-Based DESs (CholCl:EG and CholCl:Urea) at Various Weight Percentages in Water Mixture. Phys. Chem. Chem. Phys..

[49] Dai Y., Witkamp G.J., Verpoorte R., Choi Y.H. (2015). Tailoring Properties of Natural Deep Eutectic Solvents with Water to Facilitate Their Applications. Food Chem..

[50] El Achkar T., Greige-Gerges H., Fourmentin S. (2021). Basics and Properties of Deep Eutectic Solvents: A Review. Environ. Chem. Lett..

[51] Pandey A., Rai R., Pal M., Pandey S. (2014). How Polar Are Choline Chloride-Based Deep Eutectic Solvents?. Phys. Chem. Chem. Phys..

[52] Pandey A., Pandey S. (2014). Solvatochromic Probe Behavior within Choline Chloride-Based Deep Eutectic Solvents: Effect of Temperature and Water. J. Phys. Chem. B.

[53] Patra A., Khokhar V., Pandey S. (2024). Unprecedented High Probe-Reported Polarity of Deep Eutectic Solvents Composed of Lanthanide Salts and Urea. Liquids.

[54] Wen Q., Chen J.X., Tang Y.L., Wang J., Yang Z. (2015). Assessing the Toxicity and Biodegradability of Deep Eutectic Solvents. Chemosphere.

[55] Radošević K., Čanak I., Panić M., Markov K., Bubalo M.C., Frece J., Srček V.G., Redovniković I.R. (2018). Antimicrobial, Cytotoxic and Antioxidative Evaluation of Natural Deep Eutectic Solvents. Environ. Sci. Pollut. Res..

[56] Filipović Tričković J., Zdolšek N., Brković S., Veljković F., Veličković S., Janković B., Valenta Šobot A., Nemoda M., Marinković J. (2025). Antibacterial Potential and Cytotoxicity Assessment of Zinc-Based Ternary Deep Eutectic Solvents: Towards Innovative Applications in Dental Medicine. Processes.

[57] Hayyan M., Looi C.Y., Hayyan A., Wong W.F., Hashim M.A. (2015). In Vitro and in Vivo Toxicity Profiling of Ammonium-Based Deep Eutectic Solvents. PLoS ONE.

[58] Juneidi I., Hayyan M., Hashim M.A. (2015). Evaluation of Toxicity and Biodegradability for Cholinium-Based Deep Eutectic Solvents. RSC Adv..

[59] Rahman M.M., Bou Alia A., Choi M., Trainel N., Krasniqi P., Desmons A., Cailleau C., Mercier-Nomé F., Faivre V., Fournier N. (2026). Evaluation of the Acute Toxicity of the Choline Chloride : Urea Deep Eutectic Solvent and Its Impact on the Intestinal Microbiota. Green Chem..

[60] Hayyan M., Hashim M.A., Hayyan A., Al-Saadi M.A., AlNashef I.M., Mirghani M.E.S., Saheed O.K. (2013). Are Deep Eutectic Solvents Benign or Toxic?. Chemosphere.

[61] Liu K., Li X., Shi R., Yang C., Li D. (2025). Preparation and Antibacterial Activity of Silver Nanoparticles in Deep Eutectic Solvent. J. Mol. Liq..

[62] Tang C., Huang J., Yang Y., Ye J., Liu Y., Wang J., Chen Q.J. (2025). Regulating Starch Hydrogen Bond Network: Ternary Deep Eutectic Solvents Induce Performance Enhancement and Mechanistic Insights in Starch Films. Food Res. Int..

[63] Ahmed S., Bojilov D., Exner G., Dagnon S., Manolov S., Ivanov I. (2026). Solvatochromic Polarity, Physicochemical Properties, and Spectral Analysis of New Triple NADES-Based on Urea–Glycerol. Molecules.

[64] Zdanowicz M. (2023). Influence of Urea Content in Deep Eutectic Solvents on Thermoplastic Starch Films’ Properties. Appl. Sci..

[65] Zaib Q., Eckelman M.J., Yang Y., Kyung D. (2022). Are Deep Eutectic Solvents Really Green?: A Life-Cycle Perspective. Green Chem..

[66] Zaib Q., Jeong D.H., Eckelman M.J., Kyung D. (2023). Deep Eutectic Solvents Reduce the Life Cycle Environmental Impacts and Energy Demands of Carbon Dioxide Capture. ACS Sustain. Chem. Eng..

[67] Deka B., Chakravorty P., Das A.B. (2024). Impact of Different Natural Deep Eutectic Solvents on Dissolution Behaviour and Eutectogel Structure of Jackfruit Seed Starch. J. Polym. Environ..

[68] Suflet D.M., Chitanu G.C., Popa V.I. (2006). Phosphorylation of Polysaccharides: New Results on Synthesis and Characterisation of Phosphorylated Cellulose. React. Funct. Polym..

[69] Immergut E.H., Mark H.F., Platzer N.A.J. (1965). Principles of Plasticization. Plasticization and Plasticizer Processes.

[70] Alhanish A., Abu Ghalia M. (2021). Developments of Biobased Plasticizers for Compostable Polymers in the Green Packaging Applications: A Review. Biotechnol. Prog..

[71] Viñas-Ospino A., Anticona M., Esteve M.J., Blesa J., Mizielińska M., Zdanowicz M. (2025). Poly(Butylene Succinate) Films Modified with Functional Deep Eutectic Solvent/Orange Peel Extract Systems. Food Packag. Shelf Life.

[72] Aşgın Uzun Ş., Dağdelen A.F., Gümüş Ö.Y., Dündar A.N., Sarıcaoğlu F.T. (2025). Menthol and Organic Acid-Based Hydrophobic Deep Eutectic Solvents as Plasticizers in Biodegradable Poly (Lactic Acid) Films. Food Packag. Shelf Life.

[73] Qadeer A., Anis M., Warner G.R., Potts C., Giovanoulis G., Nasr S., Archundia D., Zhang Q., Ajmal Z., Tweedale A.C. (2024). Global Environmental and Toxicological Data of Emerging Plasticizers: Current Knowledge, Regrettable Substitution Dilemma, Green Solution and Future Perspectives. Green Chem..

[74] Aichholzer W., Fritz H.G. (1998). Rheological Characterization of Thermoplastic Starch Materials. Starch/Staerke.

[75] Corradini E., De Medeiros E.S., Carvalho A.J.F., Curvelo A.A.S., Mattoso L.H.C. (2006). Mechanical and Morphological Characterization of Starch/Zein Blends Plasticized with Glycerol. J. Appl. Polym. Sci..

[76] Baran A., Fričová O., Vrábel P., Popovič Ľ., Peidayesh H., Chodák I., Hutníková M., Kovaľaková M. (2022). Effects of Urea and Glycerol Mixture on Morphology and Molecular Mobility in Thermoplastic Starch/Montmorillonite-Type Nanofiller Composites Studied Using XRD and NMR. J. Polym. Res..

[77] Wang J., Cheng F., Zhu P. (2014). Structure and Properties of Urea-Plasticized Starch Films with Different Urea Contents. Carbohydr. Polym..

[78] Zhu F. (2026). Starch Processing in Deep Eutectic Solvents: Structure, Properties, Modifications and Applications. Food Hydrocoll..

[79] Tang C., Zhou S., Mao H., Huang J., Yuan Y., Wan J., Chen Q.J. (2026). Multifunctional Roles of Deep Eutectic Solvents in Starch-Based Materials: From Dissolution to Modification and Plasticization. Starch/Staerke.

[80] Biswas A., Shogren R.L., Stevenson D.G., Willett J.L., Bhowmik P.K. (2006). Ionic Liquids as Solvents for Biopolymers: Acylation of Starch and Zein Protein. Carbohydr. Polym..

[81] Shamsuri A.A., Kuang Abdullah D. (2010). Protonation and Complexation Approaches for Production of Protic Eutectic Ionic Liquids. J. Phys. Sci..

[82] Zdanowicz M., Spychaj T. (2011). Ionic Liquids as Starch Plasticizers or Solvents. Polimery.

[83] Abbott A.P., Ballantyne A.D., Conde J.P., Ryder K.S., Wise W.R. (2012). Salt Modified Starch: Sustainable, Recyclable Plastics. Green Chem..

[84] Leroy E., Decaen P., Jacquet P., Coativy G., Pontoire B., Reguerre A.L., Lourdin D. (2012). Deep Eutectic Solvents as Functional Additives for Starch Based Plastics. Green Chem..

[85] Abbott A.P., Abolibda T.Z., Davis S.J., Emmerling F., Lourdin D., Leroy E., Wise W.R. (2014). Glycol Based Plasticisers for Salt Modified Starch. RSC Adv..

[86] Ramesh S., Shanti R., Morris E. (2012). Exerted Influence of Deep Eutectic Solvent Concentration in the Room Temperature Ionic Conductivity and Thermal Behavior of Corn Starch Based Polymer Electrolytes. J. Mol. Liq..

[87] Ramesh S., Shanti R., Morris E. (2012). Studies on the Plasticization Efficiency of Deep Eutectic Solvent in Suppressing the Crystallinity of Corn Starch Based Polymer Electrolytes. Carbohydr. Polym..

[88] Zdanowicz M., Johansson C. (2016). Mechanical and Barrier Properties of Starch-Based Films Plasticized with Two- or Three Component Deep Eutectic Solvents. Carbohydr. Polym..

[89] Zdanowicz M., Johansson C. (2017). Impact of Additives on Mechanical and Barrier Properties of Starch-Based Films Plasticized with Deep Eutectic Solvents. Starch-Stärke.

[90] Favero J., Belhabib S., Guessasma S., Decaen P., Reguerre A.L., Lourdin D., Leroy E. (2017). On the Representative Elementary Size Concept to Evaluate the Compatibilisation of a Plasticised Biopolymer Blend. Carbohydr. Polym..

[91] Adamus J., Spychaj T., Zdanowicz M., Jędrzejewski R. (2018). Thermoplastic Starch with Deep Eutectic Solvents and Montmorillonite as a Base for Composite Materials. Ind. Crops Prod..

[92] Grylewicz A., Spychaj T., Zdanowicz M. (2019). Thermoplastic Starch/Wood Biocomposites Processed with Deep Eutectic Solvents. Compos. Part A Appl. Sci. Manuf..

[93] Zdanowicz M., Sałasińska K., Lewandowski K., Skórczewska K. (2022). Thermoplastic Starch/Ternary Deep Eutectic Solvent/Lignin Materials: Study of Physicochemical Properties and Fire Behavior. ACS Sustain. Chem. Eng..

[94] Wilpiszewska K., Skowrońska D. (2023). Evaluation of Starch Plasticization Efficiency by Deep Eutectic Solvents Based on Choline Chloride. J. Mol. Liq..

[95] Zdanowicz M., Sałasińska K. (2023). Characterization of Thermoplastic Starch Plasticized with Ternary Urea-Polyols Deep Eutectic Solvent with Two Selected Fillers: Microcrystalline Cellulose and Montmorillonite. Polymers.

[96] Zdanowicz M., Rokosa M., Pieczykolan M., Antosik A.K., Chudecka J., Mikiciuk M. (2023). Study on Physicochemical Properties of Biocomposite Films with Spent Coffee Grounds as a Filler and Their Influence on Physiological State of Growing Plants. Int. J. Mol. Sci..

[97] Zdanowicz M., Rokosa M., Pieczykolan M., Antosik A.K., Skórczewska K. (2024). Biocomposites Based on Wheat Flour with Urea-Based Eutectic Plasticizer and Spent Coffee Grounds: Preparation, Physicochemical Characterization, and Study of Their Influence on Plant Growth. Materials.

[98] Van Nguyen C., Pham M.T., Vu T.X., Hoang P.T. (2025). Amine-rich Deep Eutectic Solvents for Cassava Thermoplastic Starch: Composition-dependent Structure–Property Mapping and Aging Resistance. J. Mater. Sci. Mater. Eng..

[99] Tang C., Huang J., Luan P., Zhang B., Wang J., Zhang Y.-F., Chen Q.-J. (2026). Multifunctional Ammonia-Responsive Starch Composites Engineered by Deep Eutectic Solvent-Cellulose Synergy and Antimicrobial Cu-MOF for Intelligent Food Tags. Food Res. Int..

[100] Tang C., Cao J., Zhu Y., Wang N., Tian X., Chen Q.-J. (2026). Deep Eutectic Solvent–Regulated Starch Films Integrated with CuZn-MOF for Ammonia-Responsive and Visual Food Freshness Monitoring. Int. J. Biol. Macromol..

[101] Zdanowicz M., Jędrzejewski R., Pilawka R. (2019). Deep Eutectic Solvents as Simultaneous Plasticizing and Crosslinking Agents for Starch. Int. J. Biol. Macromol..

[102] Paluch M., Ostrowska J., Tyński P., Sadurski W., Konkol M. (2022). Structural and Thermal Properties of Starch Plasticized with Glycerol/Urea Mixture. J. Polym. Environ..

[103] Ivanič F., Jochec-Mošková D., Janigová I., Chodák I. (2017). Physical Properties of Starch Plasticized by a Mixture of Plasticizers. Eur. Polym. J..

[104] Šoltýs A., Hronský V., Šmídová N., Olčák D., Ivanič F., Chodák I. (2019). Solid-State 1H and 13C NMR of Corn Starch Plasticized with Glycerol and Urea. Eur. Polym. J..

[105] Zdanowicz M., Staciwa P., Jędrzejewski R., Spychaj T. (2019). Sugar Alcohol-Based Deep Eutectic Solvents as Potato Starch Plasticizers. Polymers.

[106] Acharya S., Liyanage S., Abidi N., Parajuli P., Rumi S.S., Shamshina J.L. (2021). Utilization of Cellulose to Its Full Potential: A Review on Cellulose Dissolution, Regeneration, and Applications. Polymers.

[107] Medronho B., Lindman B. (2015). Brief Overview on Cellulose Dissolution/Regeneration Interactions and Mechanisms. Adv. Colloid Interface Sci..

[108] Laus G., Bentivoglio G., Schottenberger H., Kahlenberg V., Kopacka H., Röder T., Sixta H. (2005). Ionic Liquids: Current Developments, Potential and Drawbacks for Industrial Applications. Lenzing. Berichte.

[109] Wang S., Zhang L., Khan K.A., Ullah M.W., Fan Y., Wang Z. (2025). Deep Eutectic Solvents as a ‘Multifunctional Operating Platform’ for Nanocellulose Production and Modification: A Review on Sustainable Composite Advancements. Mater. Sci. Eng. R Rep..

[110] Wang S., Peng X., Zhong L., Jing S., Cao X., Lu F., Sun R. (2015). Choline Chloride/Urea as an Effective Plasticizer for Production of Cellulose Films. Carbohydr. Polym..

[111] Wei X., Lin T., Du H., Wang L., Yin X. (2022). Effect of a Trace Amount of Deep Eutectic Solvents on the Structure and Optical Properties of Cellulose Nanocrystal Films. Cellulose.

[112] Feng Y., Li L., Miao G., Guo Q., Zhang M., Zhang X., Xu F. (2025). Nonderivatized Regenerated Cellulose Film with Plastic-Like Elongation from TMAH·5H2O/Urea. ACS Sustain. Chem. Eng..

[113] Ramesh S., Shanti R., Morris E. (2013). Characterization of Conducting Cellulose Acetate Based Polymer Electrolytes Doped with “Green” Ionic Mixture. Carbohydr. Polym..

[114] Shamsuri A.A., Daik R. (2012). Plasticizing Effect of Choline Chloride/Urea Eutectic-Based Ionic Liquid on Physicochemical Properties of Agarose films. BioResources.

[115] Shamsuri A.A., Daik R. (2013). Utilization of an Ionic Liquid/Urea Mixture as a Physical Coupling Agent for Agarose/Talc Composite Films. Materials.

[116] Sousa A.M.M., Souza H.K.S., Latona N., Liu C.K., Gonçalves M.P., Liu L. (2014). Choline Chloride Based Ionic Liquid Analogues as Tool for the Fabrication of Agar Films with Improved Mechanical Properties. Carbohydr. Polym..

[117] Sousa A.M.M., Souza H.K.S., Liu L.S., Gonçalves M.P. (2015). Alternative Plasticizers for the Production of Thermo-Compressed Agar Films. Int. J. Biol. Macromol..

[118] Wong C.Y., Wong W.Y., Walvekar R., Loh K.S., Khalid M., Lim K.L. (2018). Effect of Deep Eutectic Solvent in Proton Conduction and Thermal Behaviour of Chitosan-Based Membrane. J. Mol. Liq..

[119] Ashraf J., Zhang J., Tufail T., Awais M., Liu S., Qi Y., Ahmed Z., Yue L., Sumei Z., Xu B. (2026). Deep Eutectic Solvent: A Promising Way to Enhance the Physicomechanical and Antimicrobial Properties of Pullulan/Carboxymethyl Chitosan Films Impregnated with Zein–Nisin Nanofillers for Sustainable Food Packaging. Food Hydrocoll..

[120] Yang H., Huang X., Luo B., Yu K., Lu C., Mo J., Li P., Wei J., Zhong H. (2026). An Integrated “Extraction-Film Formation” Strategy Using Natural Deep Eutectic Solvents for Fabricating Active Films in Fruit Preservation. Food Packag. Shelf Life.

[121] Kowalonek J., Hamieau M., Szydłowska-Czerniak A. (2024). Influence of Different Deep Eutectic Solvents and Plant Extracts on Antioxidant, Mechanical, and Color Properties of Alginate Film. Polymers.

[122] Wong W.Y., Wong C.Y., Rashmi W., Khalid M. (2018). Choline Chloride:Urea-Based Deep Eutectic Solvent as Additive to Proton Conducting Chitosan Films. J. Eng. Sci. Technol..

[123] Zeng Q., Wang Y., Huang Y., Ding X., Chen J., Xu K. (2014). Deep Eutectic Solvents as Novel Extraction Media for Protein Partitioning. Analyst.

[124] Wang L., Fei T., Li X., Sun Q., Liu X., Wang L. (2026). Improved Protein Extraction from Moringa Oleifera Seeds Using Deep Eutectic Solvents: Mechanistic Insights and Protein Characterization. Food Hydrocoll..

[125] Guo J., Zhao N., Zhao Y., Jin H., Sun G., Yu J., Zhang H., Yu M., Yang D., Liang Z. (2023). Effect of Deep Eutectic Solvents on the Activity and Stability of Cellulases and Pectinases. ACS Omega.

[126] Taklimi S.M., Divsalar A., Ghalandari B., Ding X., Di Gioia M.L., Omar K.A., Saboury A.A. (2023). Effects of Deep Eutectic Solvents on the Activity and Stability of Enzymes. J. Mol. Liq..

[127] Gomes I., Galamba N. (2023). Protein Stability in a Natural Deep Eutectic Solvent: Preferential Hydration or Solvent Slaving?. J. Chem. Phys..

[128] Mamashli F., Badraghi J., Delavari B., Sabbaghian M., Hosseini M., Saboury A.A. (2019). Evaluation of Versatile Peroxidase’s Activity and Conformation in the Presence of a Hydrated Urea Based Deep Eutectic Solvent. J. Solut. Chem..

[129] Sanchez-Fernandez A., Basic M., Xiang J., Prevost S., Jackson A.J., Dicko C. (2022). Hydration in Deep Eutectic Solvents Induces Non-Monotonic Changes in the Conformation and Stability of Proteins. J. Am. Chem. Soc..

[130] Kovács A., Yusupov M., Cornet I., Billen P., Neyts E.C. (2022). Effect of Natural Deep Eutectic Solvents of Non-Eutectic Compositions on Enzyme Stability. J. Mol. Liq..

[131] Qu W., Häkkinen R., Allen J., D’Agostino C., Abbott A.P. (2019). Globular and Fibrous Proteins Modified with Deep Eutectic Solvents: Materials for Drug Delivery. Molecules.

[132] Yilmaz M.T., Kul E., Saricaoglu F.T., Odabas H.I., Taylan O., Dertli E. (2024). Deep Eutectic Solvent as Plasticizing Agent for the Zein Based Films. Food Packag. Shelf Life.

[133] Wu T., Dai R., Shan Z., Chen H., Woo M.W., Yi J. (2022). High Efficient Crosslinking of Gelatin and Preparation of Its Excellent Flexible Composite Film Using Deep Eutectic Solvent. Process Biochem..

[134] Uribarrena M., Cabezudo S., Núñez R.N., Copello G.J., de la Caba K., Guerrero P. (2025). Development of Smart Films Based on Soy Protein and Cow Horn Dissolved in a Deep Eutectic Solvent: Physicochemical and Environmental Assessment. Int. J. Biol. Macromol..

[135] Putranto A.W., Suhartini S., Wibisono Y., Masruchin N., Chua A.S.M., Ngoh G.C. (2025). Life Cycle Assessment of Deep Eutectic Solvent Employment for Sustainable Nanocellulose Production from Biomass: A Systematic Review. Green Chem. Lett. Rev..

[136] Türe H., Gällstedt M., Kuktaite R., Johansson E., Hedenqvist M.S. (2011). Protein Network Structure and Properties of Wheat Gluten Extrudates Using a Novel Solvent-Free Approach with Urea as a Combined Denaturant and Plasticiser. Soft Matter.

[137] Peñafiel-Alvarado J.I., Miranda-Pinzon M., Ivorra-Martinez J., Stefani P.M., Gomez-Caturla J., Balart R. (2025). The Effect of Plasticizers on Properties of Thermoplastic Rice Protein Processed by Extrusion and Injection Molding. J. Food Eng..

